# Disclosing the bioactive metabolites involved in the in vitro anthelmintic effects of salt-tolerant plants through a combined approach using PVPP and HPLC-ESI-MS^n^

**DOI:** 10.1038/s41598-021-03472-9

**Published:** 2021-12-21

**Authors:** Marta Oliveira, Caroline Sprengel Lima, Setha Ketavong, Eulogio J. Llorent-Martínez, Hervé Hoste, Luísa Custódio

**Affiliations:** 1 Centre of Marine Sciences, University of Algarve, Campus de Gambelas, 8005-139 Faro, Portugal; 2 Laboratory of Antibiotics and Chemotherapeutics, IBILCE, São Paulo State University, São José do Rio Preto, SP Brazil; 3 UMR 1225 IHAP, INRAe, 23 Chemin des Capelles, 31076 Toulouse, France; 4 Department of Physical and Analytical Chemistry, Faculty of Experimental Sciences, University of Jaén, Campus Las Lagunillas, 23071 Jaén, Spain; 5 ENVT, Université de Toulouse, 23 Chemin des Capelles, 31076 Toulouse, France

**Keywords:** Biochemistry, Biological techniques, Biotechnology, Drug discovery, Microbiology, Plant sciences

## Abstract

Strategies to reduce dependence on synthetic drugs for the treatment of gastrointestinal nematodes (GIN) infections in ruminants include the search for novel anthelmintic scaffolds on plants, yet salt-tolerant plants remain overlooked. This study aims to evaluate the in vitro anthelmintic properties of selected salt-tolerant plants against GIN, and identify the potential bioactive secondary metabolites involved. For that purpose, 80% acetone/water extracts were prepared from dried biomass of aerial organs of nine salt-tolerant plant species and tested against *Haemonchus contortus* and *Trichostrongylus colubriformis* by the Larval Exsheathment Inhibition Assay (LEIA) and Egg Hatching Inhibition Assay (EHIA). *Pistacia lentiscus, Limoniatrum monopetalum, Cladium mariscus* and *Helychrisum italicum picardi* were the most active in both GIN and life stages. To investigate the role of polyphenols in the anthelmintic activity, four selected extracts were treated with polyvinylpolypyrrolidone (PVPP), and non-treated and treated samples were further characterized by high-performance liquid chromatography with electrospray ionization mass spectrometric detection (HPLC-ESI-MS^n^). While polyphenols seem responsible for the EHIA properties, they are partially accountable to LEIA results. Several phenolics involved in the anthelmintic effects were identified and discussed. In sum, these species are rich sources of anthelmintic compounds and, therefore, are of major interest for nutraceutical and/or phytotherapeutic applications against GIN in ruminants.

## Introduction

Ruminants’ production represents an important agricultural sector in the Mediterranean basin, accounting for approximately 267 million heads of cattle, sheep and goats in 2019, according to FAOSTAT^1^. The global prevalence of gastrointestinal nematodes (GIN) represents a challenge to ruminants' production in outdoors systems of production since infections have a significant impact on animal health and welfare, performance and quality of animal products (e.g., milk), with consequent economic losses and without control, being causes of significant morbidity and mortality^2,3^. *Haemonchus contortus*, *Teladorsagia circumcincta*, *Trichostrongylus* spp, and *Nematodirus* spp. are the major relevant GIN species in Europe^4^. For the last 70 years, the control of GIN has relied mostly on the repeated administration of single or combinations of synthetic anthelmintic drugs, belonging to different ‘broad-spectrum” anthelmintic such as (i) benzimidazoles, (ii) levamisole, morantel, (iii) macrocyclic lactones, and (iv) monepantel (AAD)^5^. However, resistances to the different drug families are nowadays reported worldwide against different GIN species in different ruminants’ species^6^. There is also an increasing number of references on GIN populations presenting multi-resistance to several anthelmintic families. These results have encouraged the pursuit of novel sustainable and alternatives for a more integrated control with reduced reliance on synthetic anthelmintic treatments.

Plants and their bioactive products stand out as one of these non-chemical sustainable approaches to counteract GIN infections^7^. The anthelmintic properties of legume forage with containing polyphenols, including bird foot trefoil (*Lotus corniculatus* L.), big trefoil (*L. pedunculatus* Cav.)*,* sulla *(Hedysarum coronarium* L.^8,9^, and sainfoin (*Onybrichis viciifolia* Scop.)^10^, inspired further research on similar effects among other botanical groups, that could be used as nutraceuticals, but also that may represent potential sources of novel phytotherapeutic products and active principles of pharmacological interest^7^. So far, several plant extracts, fractions, and individual compounds have been studied for their potential anthelmintic properties^11,12^. The main bioactive compounds of interest for anthelmintic activity are polyphenols, particularly condensed tannins and flavonoids, but others such as terpenoids, proteinases, and saponins have also been described^12^.

A wide number of extremophile plants, including salt-tolerant species, occur in the Mediterranean area^13^. They are adapted to harsh environmental conditions, such as high sunlight exposure, UV radiation, drought, and salinity. One of these plants' evolutionary strategies to cope with such constraints includes the production and accumulation of secondary metabolites, particularly flavonoids and tannins^14^. Additionally, former investigations reveal that many species exhibit relevant bioactive properties, like antioxidant, anti-inflammatory, and enzyme inhibitory activities^15^ with diverse applications, including in veterinary medicine. Moreover, some species have ethnoveterinary uses^16^, for example, *Pistacia lentiscus* L., which is used as antiparasitic, for the treatment of bloat, constipation, and dermatological ailments in sheep and goats^17^. Nevertheless, this group of plants is still widely unexplored in the scope of veterinary parasitology. In this context, the aims of this study were (1) to evaluate the in vitro anthelmintic properties of selected Mediterranean salt-tolerant plant species against L3 larvae exsheathment and egg hatching processes of *H. contortus* and *T. colubiformis*; (2) to explore the overall role of polyphenols in the anthelmintic activity, and (3) to compare the phytochemical composition determined by high-performance liquid chromatography with electrospray ionization mass spectrometric detection (HPLC-ESI-MS^n^) of selected extracts, treated or not with poly-(poly)vinylpolirrilodine (PVPP), a polyphenol-binding agent.

## Material and methods

### Plant collection and processing

**Figure 1:**
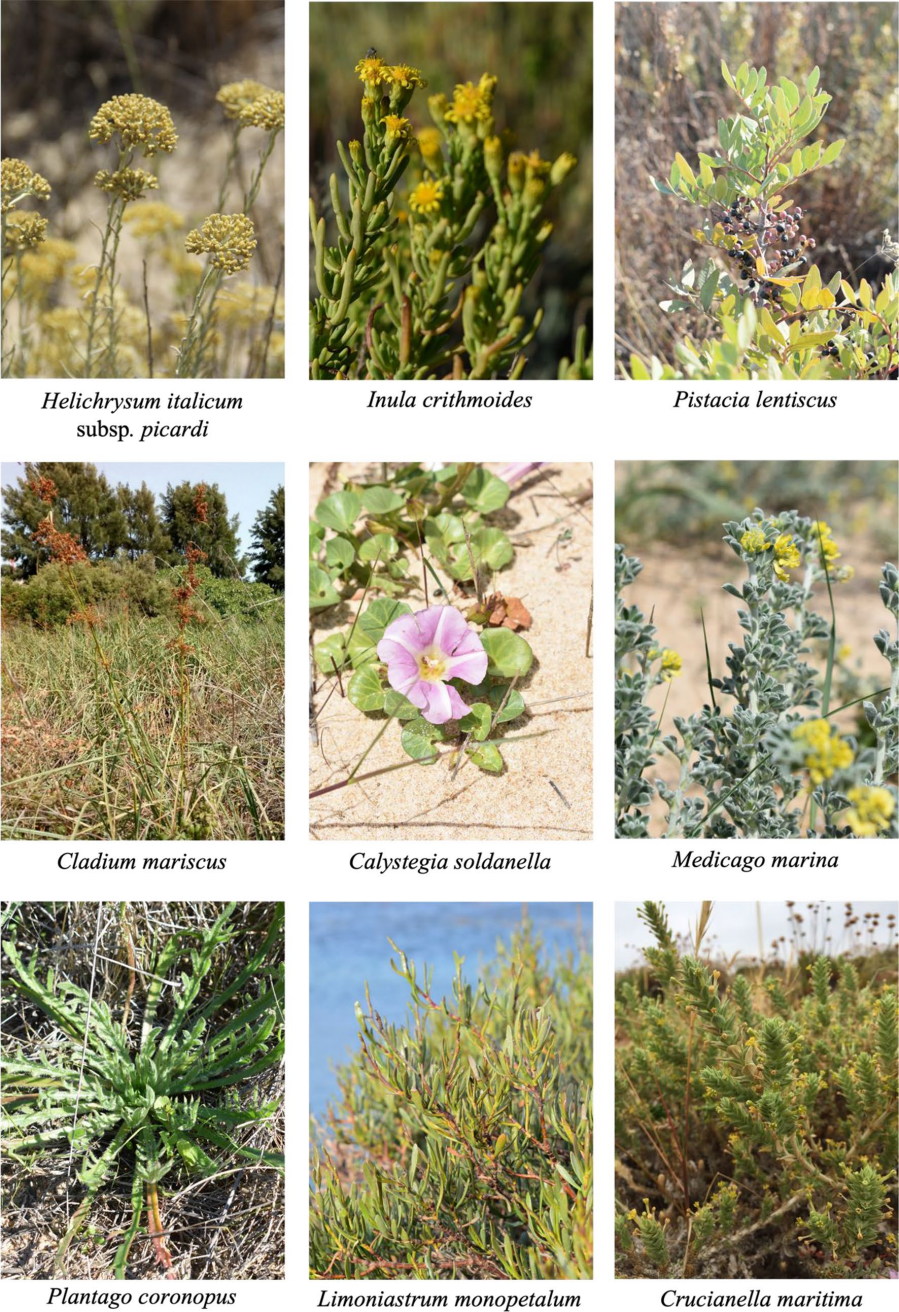
Salt-tolerant species prospected from the Algarve region, Southern Portugal.

Plant species were selected based on the ethnopharmacological uses, phenolic content reported in the literature, availability/accessibility of the biomass, and/or unreported anthelmintic properties. Thus, aerial parts of *Pistacia lentiscus* L. (Anacardiaceae), *Cladium mariscus* (L.) Pohl (Cyperaceae), *Inula crithmoides* L. (Asteraceae), *Helichrysum italicum* (Roth) G. Don subsp. *picardi* (Boiss. & Reut.) Franco (Asteraceae), *Calystegia soldanella* (L.) R. Br. (Convolvulaceae), *Medicago marina* L. (Fabaceae), *Plantago coronopus* L. (Plantaginaceae), *Limoniastrum monopetalum* (L.) Boiss. (Plumbaginaceae), and *Crucianella maritima* L. (Rubiaceae; Fig. 1) were collected in 4 districts of the Algarve coastal region (Southern Portugal), between 2017 and 2018 (Table 1). *Inula crithmoides*, *C. soldanella*, *M. marina*, *P. coronopus*, and *L. monopetalum* are halophyte plants included in the eHALOPH database^18^ while others such as *P. lentiscus*, *C. mariscus*, and *C. maritima* have recognized salt-tolerance despite not yet included in this database. After collection, samples were taken to the laboratory, washed, frozen at − 20 °C, freeze-dried (Lyoalfa 15) for three days, and ground using a coffee and a ball miller (Retsch PM 100).

**Table 1:** Plant collection details, including collected organs, date, location and voucher number.

Species/family	Voucher No.	Aerial organs	Date	Location/coordinates
*Helichrysum italicum* subsp. *picardi* (Asteraceae)	XBH32	L/FL	Jul 2017	Tavira (37° 07′ 51.8″ N, 7° 36′ 37.6″ W)
*Inula crithmoides* (Asteraceae)	XBH04	L/S/FL	Oct 2017	Olhão (37° 01′ 11.7″ N, 7° 53′ 04.8″ W)
*Pistacia lentiscus* (Anacardiaceae)	XBH06	L/S/FR	Jan 2018	Portimão (37° 07′ 34.7″ N, 8° 36′ 02.3″ W)
*Cladium mariscus* (Cyperaceae)	XBH03	L/I	Jul 2017	Faro (37° 01′ 03.3″ N,7° 59′ 18.1″ W)
*Calystegia soldanella* (Convolvulaceae)	XBH07	L/S/FL	Apr 2018	Portimão (37° 07′ 23.1″ N, 8° 36′ 10.7″ W)
*Medicago marina* (Fabaceae)	XBH41	L/S/FL	Apr 2018	Portimão (37° 07′ 23.1″ N, 8° 36′ 10.7″ W)
*Plantago coronopus* (Plantaginaceae)	XBH02	L	Jan 2018	Olhão (37° 01′ 32.8″ N, 7° 53′ 04.4″ W)
*Limoniastrum monopetalum* (Plumbaginaceae)	XBH05	L/S	Jul 2017	Portimão (37° 07′ 34.7″ N, 8° 36′ 02.3″ W)
*Crucianella maritima* (Rubiaceae)	XBH40	L/S	Apr 2017	Portimão (37° 07′ 23.2″ N, 8° 36′ 12.3″ W)

*Aerial organs*: *L* leaves, *S* stems, *FR* fruits, *FL* flowers, *I* inflorescences.

Mandatory licenses for the collection of all plant specimens from the wild in the Portuguese territory were obtained, and the collection protocol was performed according to the standard procedures recommended by “Instituto da Conservação da Natureza e das Florestas (ICNF)”, the national regulatory body. The formal identification of the plant material was made by Dr. Luísa Custódio (CCMAR). Voucher specimens were kept in the XtremeBio group herbarium, at Centre of Marine Sciences (CCMAR), University of Algarve (UAlg), Faro, Portugal (Table 1).

### Sample preparation

Dried ground samples were extracted with an 80% aqueous acetone solution (1:40, *w/v*), as previously used for the successful extraction of phenolic compounds and tannins from different salt-tolerant species^19^, at 20–25 °C, for 16 h, under stirring. Afterwards, the residue was filtered using a qualitative filter (Whatman nº 4), and acetone was removed using a rotary evaporator under reduced pressure and temperature (approximately 40 °C). The residue was later freeze-dried and recovered to be used in the in vitro anthelmintic assays.

### Phenolic content of the extracts

#### Total phenolic content (TPC)

The TPC of the extracts was estimated using the Folin–Ciocalteau (F–C) reagent^20^. Briefly, 5 µL of the extracts (10 mg mL^−1^) were added with 100 µL of the F–C reagent (1:10 in water, *v/v*) in 96-well plates, and left for 10 min at 20–25 °C, protected from light. After, it was added 100 µL of sodium carbonate (75 g L^−1^, in water) and the plate incubated for 90 min in the dark. Absorbance was measured at 725 nm in a multiplate spectrophotometer reader (Biotek Synergy 4). A calibration curve was prepared using gallic acid as a standard. TPC was expressed as gallic acid equivalents (GAE; mg GAE g extract^−1^, dry weight (DW)).

#### Total flavonoid content (TFC)

TFC was determined by the aluminum chloride (AlCl_3_) method^21^, by mixing 50 µL of the extracts at 10 mg mL^−1^ with 50 µL of 2% AlCl_3_ in methanol and left to incubate for 10 min at 20–25 °C Absorbance was measured at 415 nm in a multiplate spectrophotometer reader. A calibration curve was prepared using quercetin as a standard. TFC was expressed as quercetin equivalents (QE; mg QE g extract^−1^, DW).

#### Condensed tannins content (CTC)

CTC was evaluated by the 4-dimethylaminocinnamaldehyde-hydrochloric acid (DMACA–HCl) colorimetric method^22^ adapted to 96-well microplates^23^. Ten microliters of the extracts (10 mg mL^−1^) were mixed with 200 µL of a methanol solution of DMACA (1%, *w/v*), and 100 µL of hydrochloric acid (37%, *v/v*). After a 15 min incubation period, absorbance was measured at 640 nm in a multiplate spectrophotometer reader. A calibration curve was prepared using catechin as a standard. The concentration of CT was expressed as catechin equivalents (mg CE g extract^−1^, DW).

#### Chemical profiling by high-performance liquid chromatography with electrospray ionization mass spectrometric detection (HPLC-ESI-MS^n^)

HPLC-ESI-MS^n^ analyses were performed with an Agilent Series 1100 HPLC system with a G1315B diode array detector (Agilent Technologies), and an ion trap mass spectrometer (Esquire 6000, Bruker Daltonics) with an electrospray interface. Separation was performed in a Luna Omega Polar C_18_ analytical column (150 × 3.0 mm; 5 µm particle size) with a Polar C_18_ Security Guard cartridge (4 × 3.0 mm), both purchased from Phenomenex. Detailed chromatographic conditions are available in Supplementary Material files. Compounds’ identification was performed by mass spectrometry data. Compounds’ quantitation was carried out by UV using analytical standards of neochlorogenic acid (320 nm), chlorogenic acid (320 nm), protocatechuic acid (280 nm), catechin (280 nm), sinapic acid (320 nm), ferulic acid (320 nm), quercetin (350 nm), apigenin (350 nm), and kaempferol (350 nm). Detection limits (3σ criterion) ranged between 0.06 and 0.15 mg L^−1^. Calibration graphs were constructed in the 0.5–100 mg L^−1^ range. Peak areas at the corresponding wavelengths were plotted versus analyte concentration. Each analytical standard was used to quantify the corresponding compounds or compounds of the same chemical family for which the exact analytical standards were not available. Repeatability (n = 10) and intermediate precision (n = 9, three consecutive days) were lower than 4 and 8%, respectively. The robustness of the chromatographic method was evaluated by recording analyte signals at ± 2 nm of the optimum wavelength and by slightly varying the percentage of the mobile phase (2% changes), observing variations lower than 5% for all the analytes concerning the optimum conditions.

### In vitro anthelmintic assays

#### *Haemonchus contortus *and *Trichostrongylus colubriformis* parasites

Third-stage larvae (L3) and eggs were obtained from faeces of monospecifically infected caprine and ovine donors, with susceptible strains of *H. contortus* and *T. colubriformis*. L3 larvae had been maintained in culture flasks for 1 month, at 4 °C, before use in the Larval Exsheathment Inhibition Assay (LEIA), while eggs were collected on the day of the Egg Hatching Inhibition Assay (EHIA), and used up to 2 h after collection^24^.

#### LEIA

LEIA was performed as previously described by Bahuaud and colleagues^25^. The extracts were diluted in phosphate-buffered saline (PBS; 0.1 M phosphate, 0.05 M NaCl, pH 7.2), and incubated with L3 larvae (approx. 800 larvae per mL) at 23 °C for 3 h. Afterwards, larvae were washed and centrifuged with PBS 3 times, and the pellet resuspended in 200 µL of PBS. To initiate the LEIA, 40 µL of the test solution was used to count the proportion of ensheathed/exsheathed larvae at 0 min. The remaining larvae (160 µL) were then subjected to an artificially induced exsheathment by exposure to a solution of Milton (2% *w/v* sodium hypochlorite, 16.5% *w/v* sodium chloride) diluted in PBS. Milton optimal concentration was determined for each batch before use in order to ensure a gradual exhsheathment process, reaching 100% exsheathment in 60 min. After 20, 40, and 60 min exposure, the number of ensheathed and exsheathed larvae were counted under a microscope (400×). Four replicates were performed for each extract concentration, and the negative control, PBS, was run in parallel. Percentage of larvae exsheathment (LE) for each replicate was calculated according to the following formula: %LE = [(number of exsheathed larvae)/(number of exsheathed + ensheathed larvae) × 100].

#### EHIA

Faeces material was filtrated using a gaze hydrophyle compress for 2 times, transferred to a 25 µm sieve, and washed with distilled water. The residue was centrifuged three times using a saline saturated solution (d = 1.2) to concentrate the eggs, and the pellets were recovered in PBS for use in the experiments. Afterwards, the eggs were quantified, plated in 48-well sterile plates (100 eggs per well), and exposed to the extracts at concentrations ranging from 5000 to 78 µg mL^−1^ in PBS. Plates were incubated at 27 °C for 48 h in the dark, and the number of larvae and eggs, in each well, was registered after microscopic counting. Six replicates were performed for each extract concentration, and the negative PBS control was run in parallel. The percentage of egg hatching (EH) for each well, was calculated according to the following formula: % EH = [(number of larvae)/(number of eggs + larvae) × 100].

### Polyvinylpolypyrrolidone (PVPP) treatment

PVPP is a polyphenol inhibitor that binds to tannins and flavonoids, removing these metabolites from the solution^26^. To ascertain the role of the polyphenols in the anthelmintic activity of the extracts, PVPP was added at a ratio of 50:1 to the active ones, respectively for eggs and larvae assays, in PBS, and incubated overnight at 4 °C. The maximum concentration tested for LEIA was 1200 µg mL^−1^; for EHIA it was 2500 µg mL^−1^. Thereafter, the samples were centrifuged for 10 min at 4500 rpm, and the supernatant depleted in polyphenols was tested in LEIA and EHIA assays. The extracts exposed or not to PVPP plus a negative control (PBS) were run in parallel.

### Statistical analyses

At least four replicates per concentration were included in all experiments. The results on phenolic content are expressed as mean ± standard error of the mean (SEM). Anthelmintic data are expressed as the concentrations inhibiting 50% of larval exsheathment or egg hatching (IC_50_ values, µg mL^−1^), and 95% confidence intervals (CI), obtained by Probit analysis. SPSS Statistics v. 26.0 software was used to assess significant differences among IC_50_ values, through relative median potency estimates, and among phenolic data, by one-way analysis of variance (ANOVA) followed by the post-hoc Tukey HSD test. Spearman correlations were calculated between the total flavonoids, total phenols, and the IC_50_ values for LEIA on the 2 nematode species.

## Results and discussion

### Total phenolics, total flavonoids and condensed tannins contents

The phenolic contents of the extracts are presented in Table 2. The phenolic content of the extracts is presented in Table 2. The total phenolic content of all species ranged between 14.2 and 226.3 mg GAE eq. g^−1^ DW extract while the total flavonoid content ranged between 13.3 and 45.4 mg QE g^−1^ DW. Lopes and colleagues (2016) reported higher TPC values for *C. mariscus* (254 mg GAE g^−1^ DW), *C. soldanela* (144 mg GAE g^−1^ DW), *I. crithmoides* (141 mg GAE g^−1^ DW), *L. monopetalum* (248 mg GAE g^−1^ DW) 80% acetone water extracts, except for *P. lentiscus* (130 mg g^−1^ DW), but lower flavonoid contents in comparison to our work (1.26–13.8 mg rutin g^−1^ DW)^15^. In another work, *H. italicum picardi* infusions and decoctions of aerial organs have been previously described as rich sources of flavonoids (91.8–119 mg rutin 200 mL^−1^)^27^. Moreover, a lower combined TPC value was detected in *P. coronopus* leaves and flowers extracts of different polarities (72.1 mg GAE g^−1^ DW) but increased TFC levels (282.8 mg rutin g^−1^ DW)^28^. In this study, total condensed tannins were detected only in three species, in the following concentration order: *P. lentiscus* > *L. monopetalum* > *C. mariscus*. In agreement, tannins were formerly detected in the same three formerly mentioned species, although at lower concentrations (6.63–38.7 mg CE g^−1^ DW, extract)^15^. It is worth to mention that dissimilarities between our results and those of other authors may be the reflection of different extraction methodologies and standards employed as well as environmental and plant-related factors.

**Table 2:** Phenolic content of acetone water extracts of selected plant species.

Species	TPC	TFC	CTC
*Helichrysum italicum* subsp. *picardi*	83.7 ± 0.6^e^	45.4 ± 1.3ª	n.d.
*Inula crithmoides*	27.2 ± 1.1^g^	13.3 ± 0.1^g^	n.d.
*Pistacia lentiscus*	226.3 ± 0.8^a^	28.9 ± 0.4^c^	607.3 ± 29.4^a^
*Calystegia soldanella*	73.2 ± 0.8^f^	42.0 ± 1.0^b^	n.d.
*Cladium mariscus*	112.3 ± 2.1*^c^	18.5 ± 0.4*^ef^	153.1 ± 2.2*^c^
*Medicago marina*	14.2 ± 0.5^h^	27.0 ± 0.8^cd^	n.d.
*Plantago coronopus*	160.0 ± 3.0^b^	25.2 ± 0.5^d^	n.d.
*Limoniastrum monopetalum*	96.7 ± 2.9^d^	16.0 ± 0.3^fg^	281.4 ± 22.6^b^
*Crucianella maritima*	25.5 ± 0.9^g^	20.4 ± 0.2^e^	n.d.

*n.d.* not detected, *TPC* total phenolic content, expressed as mg gallic acid equivalents g^−1^ extract (mg GAE g^−1^, DW), *TFC* total flavonoid content, expressed as mg quercetin equivalents g^−1^ extract (mg QE g^−1^, DW), *CTC* condensed tannins content, expressed as mg catechin equivalents g^−1^ extract (mg CE g^−1^, DW). Values are expressed as mean with standard deviation of the mean represented. *Data published in^49^. Different letters superscript represent significant differences among species, for each assay (*p* < 0.05; Tukey HSD).

### In vitro anthelmintic properties

Table 3 summarizes the results of the in vitro activity of salt-tolerant plant extracts against *H. contortus* L3 larvae and eggs and *T. colubriformis* L3 larvae and eggs obtained in LEIA and EHIA assays. Lentisk (*P. lentiscus*) exhibited the highest activity on LEIA (IC_50_ = 27.8–29.7 µg mL^−1^) and egg hatching processes (IC_50_ = 197.7 and 223.9 µg mL^−1^), without significant differences between GIN species. Lentisk is an evergreen shrub with high polyphenol content and previous results have shown both in vitro and in vivo anthelmintic properties^29–32^. In previous studies, *P. lentiscus* extracts (acetone, ethanol and/or water) exhibited less than 20% larvae exsheathment and migration at 1200 µg mL^−1^^29,31^. Nevertheless, the results for the in vitro egg hatching assay are herein, to the best of our knowledge, described for the first time.

**Table 3:** In vitro anthelmintic activity of acetone extracts of selected plants on *H. contortus* and *T. colubriformis*, by L3 larvae exsheathment (LEIA) and egg hatching assays (EHIA).

Species	LEIA		EHIA	
	*H. contortus*	*T. colubriformis*	*H. contortus*	*T. colubriformis*
*Helichrysum italicum* subsp. *picardi*	92.8^Ab^ (78.9–107.4)	132.5^Bcd^ (112.0–157.1)	2947.7^Ac^ (2772.5–3136.1)	3707.5^Bd^ (3494.4–3941.5)
*Inula crithmoides*	300.8^Ac^ (231.5–391.2)	1030.8^Be^ (731.3–1563.0)	n.d.	n.d.
*Pistacia lentiscus*	27.8^Aa^ (21.3–36.8)	29.7^Aa^ (22.2–39.7)	197.7^Aa^ (158.3–243.8)	223.9^Aa^ (185.0–268.7)
*Calystegia soldanella*	270.6^Ac^ (204.9–368.2)	270.8^Ad^ (197.7–384.4)	n.d.	n.d.
*Cladium mariscus*	88.9^Ab^ (66.3–118.7)	77.8^Abc^ (60.6–100.0)	1496.6^Ab^ (1326.5–1698.9)	2575.5^Bc^ (2324.1–2881.8)
*Medicago marina*	222.6^Ac^ (179.8–278.6)	211.2^Ad^ (159.7–282.2)	n.d.	3860.5^d^ (3501.6–4343.8)
*Plantago coronopus*	94.0^Ab^ (71.6–121.2)	212.4^Bd^ (156.3–292.6)	n.d.	n.d.
*Limoniastrum monopetalum*	39.4^Aa^ (33.2–46.4)	47.9^Aab^ (37.1–60.4)	1999.9^Ab^ (1693.6–2408.2)	2102.5^Ab^ (1813.2–2477.8)
*Crucianella maritima*	447.2^Ad^ (302.5–707.7)	1024.5^Be^ (616.9–2153.1)	n.d.	n.d.

Results are expressed as IC_50_ values (µg mL^−1^) and 95% confidence intervals in brackets.

*n.d.* not determined since IC_50_ is higher than 5000 µg mL^−1^. Capital and small letters represent significant statistical differences among botanical species (rows) and GIN species (columns) for each assay, respectively, based on Relative Median Potency Estimates.

Following *P. lentiscus*, *L. monopetalum, C. mariscus* and *H. italicum. picardi* extracts exhibited the most promising results towards both GIN species and life stages (Table 3). *Limoniastrum monopetalum* is a highly salt-tolerant shrub, widely distributed in the Mediterranean area, and was as effective as *P. lentiscus* in LEIA (*p* < 0.05), with IC_50_ values lower than 50 µg mL^−1^ (no significant difference between the two tested parasites; *p* > 0.05). In EHIA, *L. monopetalum* was also the most active species, besides *P. lentiscus*, with similar activity towards both parasites (IC_50_ = 1999.9 and 2102.5 µg mL^−1^, respectively). *Cladium mariscus*, or sawgrass, is an evergreen grass-like plant occurring in coastal saltmarshes in the Mediterranean region. *C. mariscus* extract inhibited L3 larvae exsheathment (IC_50_ = 77.8–88.9 µg mL^−1^), without significant differences between both parasite species (*p* > 0.05). In contrast, in the EHIA, *C. mariscus* was more effective towards *H. contortus* (IC_50_ = 1496.6 µg mL^−1^) than *T. colubriformis* (IC_50_ = 2575.5 µg mL^−1^; *p* < 0.05). *Helichrysum italicum* subsp. *picardi* (everlasting) is an aromatic salt tolerant plant commonly found in sandy soils, such as sand dunes, along the Southern European coast. Everlasting extract exhibited IC_50_ values ranging between 92.8–132.5 µg mL^−1^ on LEIA, and 2947.7–3707.5 µg mL^−1^ on EHIA. Interestingly, *H. contortus* larvae and eggs were more susceptible to the *H. italicum picardi* extract than those of *T. colubriformis* (*p* < 0.05).

It is well recognized that the anthelmintic activity is affected by the class, structure and concentration of secondary metabolites^7^. Moreover, these metabolites have different effects, depending on the target parasite species and life development stages^7^. A higher susceptibility of *H. contortus* in comparison to *T. colubriformis*, as observed for *C. mariscus* and *H. italicum picardi* extracts, has been previously documented for other bioactive plants, such as sainfoin, and individual chemical structures, depending on the ratios of prodelphinidins/procyanidins^10,33,34^. The authors suggest that such differences can reflect dissimilarities on the composition of specific parasite sheath proteins, that interact differently with the chemical groups^33,34^. The same conclusion can be driven for differences among parasite stages, as the eggshell and larvae coat differ in their structural components, which has also been recorded with conventional anthelmintic drugs^7,35^. This may explain the results obtained for *P. coronopus,* which was more active against larvae exsheathment (IC_50_ = 94.0 and 212.4 µg mL^−1^), and inactive towards eggs, of both parasite species, at the maximum concentration tested. Overall, IC_50_ results obtained in LEIA are frequently reported as lower than EHA, suggesting that infective L3 larvae are more susceptible than eggs^36,37^.

*Calystegia soldanella*, *C. maritima* and *M. marina* co-occur in sand dunes along the Algarve coastline while *I. crithmoides* can be found in highly saline environments, such as saltmarshes. These four species were mildly to poorly active on both assays (Table 3). Interestingly, while *I. crithmoides* was mostly ineffective in this study, its related species, *I. viscosa* 70% ethanolic extract exhibit anthelmintic properties against the larvae exsheathment of a mixture of *Teladorsagia circumcincta* and *T. colubriformis* parasites^32^, suggesting significant chemical diversity among the genus.

Overall, the nine plant extracts had comparable effects between the two GIN species (Spearmen correlation; R^2^ = 0.96; *p* < 0.01). In addition, a negative correlation between the total phenolic content and the anthelmintic activity was noted, particularly with *H. contortus* parasites (Spearmen correlation; R^2^ = 0.783; *p* < 0.05), suggesting that these metabolites may be involved in the anti-parasitic nematode’s effects.

### Role of polyphenols in the anthelmintic activity: PVPP as a polyphenol binding agent

In order to ascertain the role of polyphenols in the anthelmintic properties, the four plant extracts presenting results for both LEIA and EHIA were selected for further studies using PVPP. PVPP is a polyphenol inhibitor, as it binds to tannins and flavonoids, removing these metabolites from the solution^26^. Thus, if after PVPP exposure a loss of the anthelmintic activity is observed, it can be assumed that polyphenols are most probably responsible for the activity once they were formerly removed.

**Figure 2:**
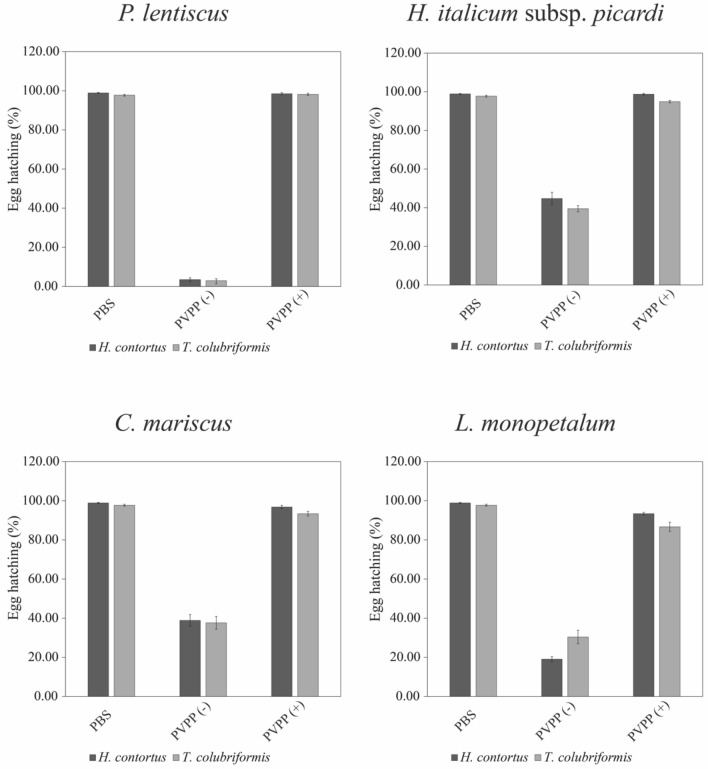
Effect of the application of PVPP on extracts of 4 selected plants, on the egg hatching inhibitory assay (EHIA) for *H. contortus* and *T. colubriformis* at concentration of 2500 µg mL^−1^, either treated [PVPP(+)] or not [PVPP(−)] with PVPP.

**Figure 3:**
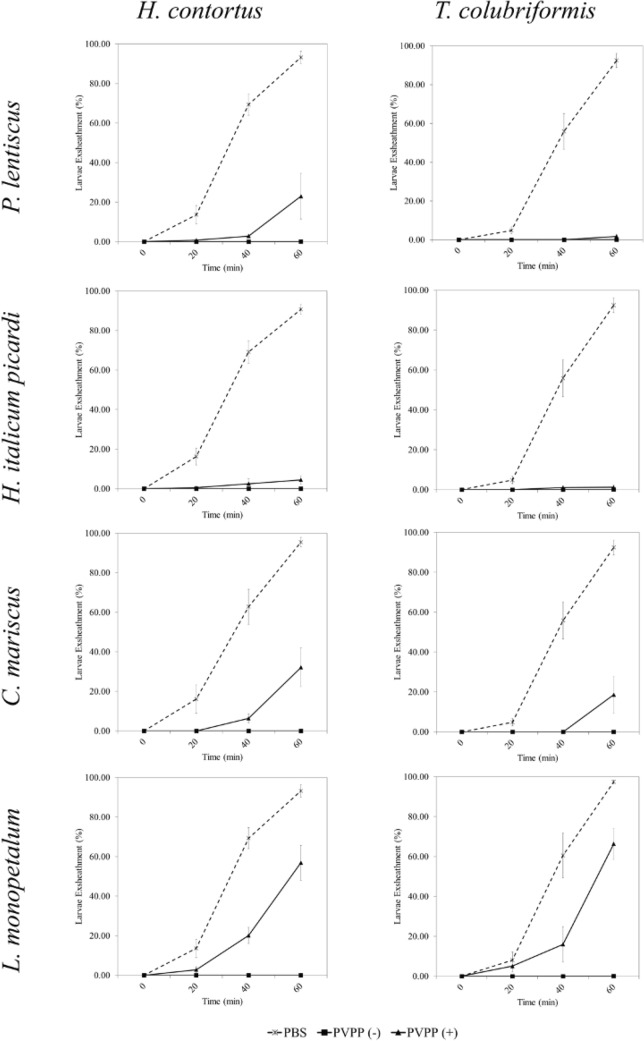
Effect of the application of PVPP on extracts of 4 selected plants, on L3 larvae exsheathment assay (LEIA) for *H. contortus* and *T. colubriformis* at concentration of 2500 µg mL^−1^, either treated [PVPP(+)] or not [PVPP(−)] with PVPP.

The effects of the addition of PVPP to extracts on EHIA and LEIA are illustrated in Figs. 2 and 3, respectively. The application of all the extracts with PVPP largely restored the egg hatching process (Fig. 2) to control values, suggesting that polyphenols are most probably involved in the inhibition of this life stage development. Vargas-Magaña and colleagues (2014), while exploring the role of polyphenols on the anthelmintic effects of several extracts of tannin-containing tropical plants on EHIA, concluded that the main mechanism of action was by impairing larvae eclosion from the eggs^38^. Likewise, we noted a high number of larvae trapped inside the eggs after the application of these active extracts (data not shown).

In contrast to EHIA, results with PVPP varied on LEIA (Fig. 3): the application of the *L. monopetalum* extract, resulted in 60–70% of larvae exsheathment of both parasite species after PVPP addition for 60 min, in contrast to 0% in the non-treated sample; the extract from *H. italicum picardi* pre-incubated with PVPP remained mostly completely active. Subtle changes were observed for *C. mariscus* (approx. 20–40% of larvae exsheathment after 60 min of treatment) for both parasite species, while *P. lentiscus* had only around 20% of larvae exsheathment at 60 min, after PVPP treatment. These results suggest that other bioactive metabolites, alone or in synergy, can be present in all extracts tested, especially for *H. italicum picardi*, *P. lentiscus*, and *C. mariscus*. In agreement with our results, other authors already reported that *P. lentiscus* extracts remain active on GIN larvae migration after exposure to PVPP^29^.

The remaining activity on LEIA for the majority of the extracts tested should be carefully analyzed, and two scientific questions arise. First, was the ratio of PVPP used insufficient to cope with the high phenolic content of the extracts? Despite being commonly used, Manoloraki et al. (2010) questioned this hypothesis when testing *P. lentiscus* for larvae migration after PVPP addition, since this species has a high polyphenol content, comparable to our results^29^. On the other hand, are other bioactive metabolites present in the extracts that are also effective in inhibiting larvae exsheathment? For instance, different authors suggest that terpenes may be responsible for the remaining in vitro and in vivo anthelmintic properties of *P. lentiscus* after the addition of PVPP or polyethylene glycol (PEG), a similar inhibitor of polyphenols^29,30^. Additionally, Botura and colleagues (2013) described that the flavonoid fraction of *Agave sisalana* Perrine (sisal) had higher activity on egg hatching, while the saponin fraction had mostly larvicidal effects^39^. In an attempt to address these scientific questions, and elucidate the possible metabolites involved, we have conducted an HPLC-ESI-MS^n^ comparative analysis on the active samples, before and after PVPP treatment.

### HPLC-ESI-MS^n^ comparative analysis of the chemical profile of non-treated and treated-PVPP samples

**Figure 4:**
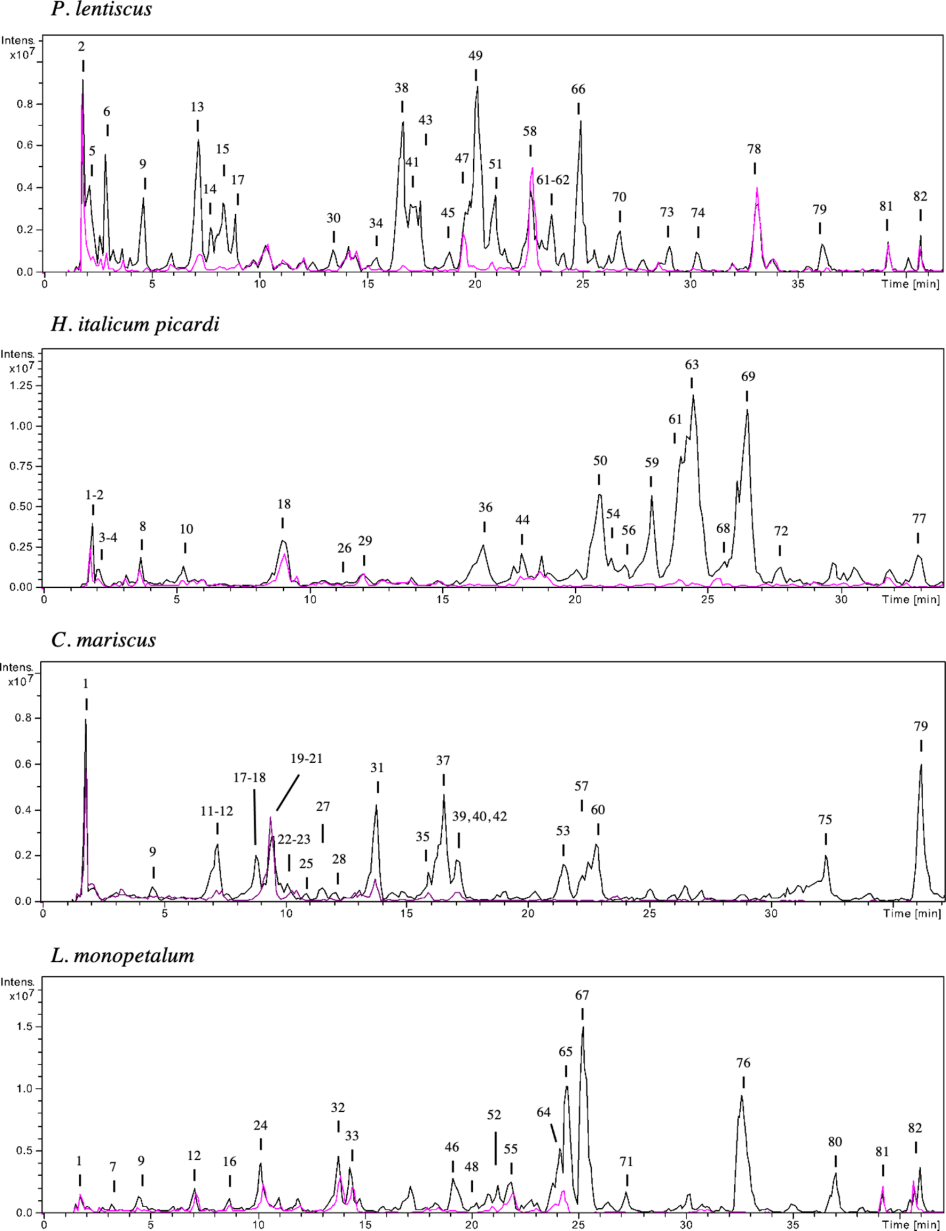
Base peak chromatogram of the extracts of 4 selected plants. The blackline represents the chromatogram of non-treated samples, while the pink line represents the chromatogram of PVPP-treated extracts, with numbers referring to the compounds described in Tables 4, 5, 6 and 7.

The HPLC-ESI-MS^n^ analysis was performed in the most active extracts, with and without PVPP. Obtained chromatograms are represented in Fig. 4 while the chemical profile of each species is depicted in Tables 4, 5, 6 and 7. The characterization of the compounds is detailed in Supplementary Material files.

**Table 4:** Chemical profile of the extract of *Pistacia lentiscus* aerial organs.

No.	Rt (min)	[M–H]^−^ m/z	m/z (% base peak)	Assigned identification	PVPP
2	1.9	191	MS2 [191]: 173 (100)	Quinic acid	+
5	2.2	495	MS2 [495]: 343 (100), 325 (14), 169 (16)	Di-*O*-Galloylquinic acid	
			MS3 [495 → 343]: 191 (99), 169 (100), 125 (20)		
			MS4 [495 → 343 → 169]: 125 (100)		
6	2.9	343	MS2 [343]: 191 (100), 169 (15), 125 (4)	Galloylquinic acid	+
9	4.6	305	MS2 [305]: 261 (31), 221 (35), 219 (71), 179 (100), 165 (38)	(Epi)gallocatechin	
13	7.2	495	MS2 [495]: 343 (100), 325 (7), 169 (13)	Di-*O*-Galloylquinic acid	
			MS3 [495 → 343]: 191 (100), 169 (77), 125 (10)		
14	7.8	495	MS2 [495]: 343 (100), 325 (36), 191 (12), 169 (15)	Di-*O*-Galloylquinic acid	
			MS3 [495 → 343]: 191 (40), 173 (9), 169 (100), 125 (10)		
15	8.4	183	MS2 [183]: 168 (100)	Methyl gallate	
			MS3 [183 → 168]: 124 (100)		
17	8.8	289	MS2 [289]: 245 (100), 205 (40), 203 (14), 179 (22), 151 (9)	Catechin	
30	13.4	457	MS2 [457]: 331 (22), 305 (21), 169 (100)	(Epi)gallocatechin gallate	
			MS3 [457 → 169]: 125 (100)		
34	15.5	631	MS2 [631]: 479 (100)	Myricetin-hexoside-gallate	
			MS3 [631 → 479]: 317 (100), 316 (93), 179 (10)		
			MS4 [631 → 479 → 317]: 271 (100), 179 (38)		
38	16.6	625	MS2 [625]: 317 (100), 316 (87)	Myricetin-*O*-rutinoside	+
			MS3 [625 → 317]: 271 (100), 179 (90), 151 (22)		
41	17.1	493	MS2 [493]: 317 (100)	Myricetin-*O*-glucuronide	
			MS3 [493 → 317]: 179 (100), 151 (29)		
43	17.5	479	MS2 [479]: 317 (100), 316 (97)	Myricetin-*O*-hexoside	
			MS3 [479 → 317]: 271 (100), 179 (66), 151 (12)		
45	18.8	615	MS2 [615]: 463 (100), 301 (42)	Quercetin-hexoside-gallate	
			MS3 [615 → 463]: 301 (100)		
			MS4 [615 → 463 → 301]: 179 (98), 151 (100)		
47	19.6	449	MS2 [449]: 317 (44), 316 (100)	Myricetin-*O*-pentoside	
			MS3 [449 → 316]: 271 (100), 179 (26)		
49	20.1	463	MS2 [463]: 317 (95), 316 (100)	Myricetin-*O*-deoxyhexoside	+
			MS3 [463 → 316]: 271 (100), 179 (80), 151 (20)		
51	20.9	463	MS2 [463]: 301 (100)	Quercetin-*O*-hexoside	
			MS3 [463 → 301]: 179 (100), 151 (50)		
58	22.6	373 (+)	MS2 [373]: 211 (100), 193 (34), 175 (16), 135 (22), 119 (14)	Hydroferuloylglucose	+
61	23.4	433	MS2 [433]: 301 (100)	Quercetin-*O*-pentoside	
			MS3 [433 → 301]: 271 (100), 179 (87), 151 (68)		
62	23.5	447	MS2 [447]: 285 (100)	Kaempferol-*O*-hexoside	
			MS3 [447 → 285]: 255 (100), 229 (37), 227 (33)		
66	24.8	447	MS2 [447]: 301 (100)	Quercetin-*O*-deoxyhexoside	+
			MS3 [447 → 301]: 179 (48), 151 (100)		
70	26.7	585	MS2 [585]: 301 (100)	Quercetin-pentoside-gallate	
			MS3 [585 → 301]: 179 (100), 151 (98)		
73	29.0	431	MS2 [431]: 285 (100)	Kaempferol-*O*-deoxyhexoside	+
			MS3 [431 → 285]: 257 (93), 255 (100), 241 (55), 229 (36)		
74	30.2	569	MS2 [569]: 285 (100)	Kaempferol-pentoside-gallate	
			MS3 [569 → 285]: 285 (100), 257 (37), 151 (86)		
78	33.0	507	MS2 [507]: 461 (100), 293 (36)	Unknown	+
79	36.0	285	MS2 [285]: 285 (100), 241 (23)	Luteolin	
81	39.1	327	MS2 [327]: 291 (24), 229 (100), 211 (25), 171 (89)	Oxo-dihydroxy-octadecenoic acid	+
82	40.6	329	MS2 [329]: 311 (31), 229 (96), 211 (100), 171 (60)	Trihydroxy-octadecenoic acid	+

Column "PVPP" indicate if the compound was also present in the corresponding extract treated with PVPP.

**Table 5:** Characterization of the compounds present in the extract of *Helichrysum italicum picardi* aerial organs.

No.	Rt (min)	[M–H]^−^ *m/z*	m/z (% base peak)	Assigned identification	PVPP
1	1.8	377	MS^2^ [377]: 341 (100)	Disaccharide (HCl adduct)	+
			MS^3^ [377 → 341]: 179 (100), 161 (95), 143 (34)		
			MS^4^ [377 → 341 → 179]: 143 (94), 119 (100)		
2	1.9	191	MS^2^ [191]: 173 (48), 111 (100)	Quinic acid	+
3	2.1	315	MS^2^ [315]: 153 (100)	Dihydroxybenzoic acid-*O*-hexoside	+
			MS^3^ [315 → 153]: 123 (100), 108 (49)		
4	2.1	353	MS^2^ [353]: 191 (100), 179 (26), 135 (7)	Caffeoylquinic acid	+
8	3.7	315	MS^2^ [315]: 153 (100)	Dihydroxybenzoic acid-*O*-hexoside	+
			MS^3^ [315 → 153]: 109 (100)		
10	5.3	353	MS^2^ [353]: 191 (100), 179 (37), 135 (9)	Neochlorogenic acid	+
18	9.0	353	MS^2^ [353]: 191 (100), 179 (4), 173 (5), 135 (3)	Chlorogenic acid	+
26	11.2	179	MS^2^ [179]: 135 (100)	Caffeic acid	
29	12.2	609	MS^2^ [609]: 447 (100), 285 (37)	Kaempferol-dihexoside	+
			MS^3^ [609 → 447]: 285 (46), 284 (100), 255 (50), 151 (20)		
			MS^4^ [609 → 447 → 285]: 255 (100), 243 (15), 227 (17)		
36	16.4	479	MS^2^ [479]: 317 (100)	Unidentified-*O*-hexoside	
			MS^3^ [479 → 317]: 317 (100), 203 (10), 195 (16), 165 (21)		
44	18.0	515	MS^2^ [515]: 353 (100), 191 (12)	Dicaffeoylquinic acid	
			MS^3^ [515 → 353]: 191 (100), 179 (44), 173 (13), 135 (13)		
50	20.8	463	MS^2^ [463]: 301 (100)	Quercetin-*O*-hexoside	
			MS^3^ [463 → 301]: 179 (24), 151 (100)		
54	21.6	493	MS^2^ [493]: 331 (100)	Mearnsetin-*O*-hexoside	
			MS^3^ [493 → 331]: 316 (100)		
56	22.2	477	MS^2^ [477]: 315 (100), 314 (16)	Isorhamnetin-*O*-hexoside	
			MS^3^ [477 → 315]: 300 (100)		
59	22.7	515	MS^2^ [515]: 353 (100), 179 (18), 173 (21)	Dicaffeoylquinic acid	
			MS^3^ [515 → 353]: 191 (48), 179 (62), 173 (100), 135 (10)		
61	23.4	433	MS^2^ [433]: 301 (100), 271 (12)	Quercetin-*O*-pentoside	
			MS^3^ [433 → 301]: 271 (68), 255 (100), 179 (18), 151 (55)		
63	24.1	515	MS^2^ [515]: 353 (100), 191 (7), 179 (3)	Dicaffeoylquinic acid	+
			MS^3^ [515 → 353]: 191 (100), 179 (58), 135 (21)		
68	25.4	431	MS^2^ [431]: 269 (100)	Apigenin-*O*-hexoside	+
			MS^3^ [431 → 269]: 225 (100)		
69	26.5	515	MS^2^ [515]: 353 (100), 179 (12), 173 (18)	Dicaffeoylquinic acid	+
			MS^3^ [515 → 353]: 191 (13), 179 (68), 173 (100), 135 (15)		
72	27.4	463	MS^2^ [463]: 301 (100)	Quercetin-*O*-hexoside	
			MS^3^ [463 → 301]: 179 (100), 151 (76)		
77	32.7	609	MS^2^ [609]: 463 (100), 301 (47)	Quercetin-*O*-deoxyhexoside-*O*-hexoside	
			MS^3^ [609 → 463]: 301 (100), 271 (4)		
			MS^4^ [609 → 463 → 301]: 179 (62), 151 (100)		

Column "PVPP" indicate if the compound was also present in the corresponding *H. italicum picardi* treated PVPP sample.

**Table 6:** Characterization of the compounds present in the extract of *Cladium mariscu*s aerial organs.

No.	Rt (min)	[M–H]^−^ *m/z*	m/z (% base peak)	Assigned identification	PVPP
1	1.8	377	MS^2^ [377]: 341 (100)	Disaccharide (HCl adduct)	+
			MS^3^ [377 → 341]: 179 (100), 161 (24), 143 (13), 119 (25), 113 (20)		
9	4.6	305	MS^2^ [305]: 261 (7), 221 (43), 219 (72), 179 (100), 165 (35)	(Epi)gallocatechin	
11	7.0	577	MS^2^ [577]: 451 (38), 425 (100), 407 (96), 305 (21), 289 (45), 287 (17)	Procyanidin dimer	
12	7.2	305	MS^2^ [305]: 261 (12), 221 (55), 219 (77), 179 (100), 165 (26)	(Epi)gallocatechin	
17	8.8	289	MS^2^ [289]: 245 (100), 205 (43), 203 (28), 179 (24)	Catechin	
18	9.0	353	MS^2^ [353]: 191 (100), 179 (3), 173 (4), 135 (1)	Chlorogenic acid*	+
19	9.3	865	MS^2^ [865]: 739 (54), 713 (41), 695 (100), 577 (52), 451 (29), 407 (54), 405 (23), 289(19), 287 (41)	Proanthocyanidin trimer	
20	9.5	429	MS^2^ [429]: 267 (100)	Unknown	+
			MS^3^ [429 → 267]: 205 (100), 113 (82)		
21	9.9	577	MS^2^ [577]: 451 (69), 441 (17), 425 (30), 305 (100), 289 (10), 287 (8)	Proanthocyanidin dimer	
22	10.1	865	MS^2^ [865]: 739 (76), 695 (100), 577 (83), 451 (18), 407 (97), 287 (58)	Proanthocyanidin trimer	
23	10.1	561	MS^2^ [561]: 543(18), 435 (58), 409 (73), 425 (46), 289 (100), 271 (41)	Proanthocyanidin dimer	
			MS^3^ [561 → 289]: 245 (100), 205 (57), 203 (30)		
25	10.9	577	MS^2^ [577]: 451 (25), 441 (9), 425 (100), 407 (61), 305 (43), 289 (33), 287 (10)	Proanthocyanidin dimer	
27	11.5	577	MS^2^ [577]: 451 (28), 425 (10), 305 (100), 289 (4), 287 (6)	Proanthocyanidin dimer	
28	12.1	289	MS^2^ [289]: 245 (100), 205 (48), 203 (19), 179 (25), 161 (10)	Epicatechin	
31	13.7	579	MS^2^ [579]: 561 (16), 519 (16), 489 (100), 459 (99), 429 (18), 399 (50), 369 (14)	Luteolin-*C*-hexoside-*C*-pentoside	+
35	15.9	563	MS^2^ [563]: 545 (14), 503 (15), 473 (48), 443 (100), 383 (37), 353 (43)	Apigenin-*C*-hexoside-*C*-pentoside	+
37	16.5	447	MS^2^ [447]: 429 (14), 357 (70), 327 (100), 285 (3)	Luteolin‐6‐*C*‐glucoside (isoorientin)	+
39	17.0	461	MS^2^ [461]: 341 (100), 313 (66), 298 (37)	Unknown	+
40	17.0	549	MS^2^ [549]: 531 (12), 489 (26), 459 (100), 441 (13), 429 (10), 399 (64), 369 (25)	Luteolin 6-*C*-pentosyl-8-*C*-pentoside	+
42	17.3	563	MS^2^ [563]: 503 (22), 473 (100), 443 (69), 383 (61), 353 (97)	Apigenin-*C*-hexoside-*C*-pentoside	+
53	21.4	447	MS^2^ [447]: 285 (100)	Kaempferol-*O*-hexoside	
			MS^3^ [447 → 285]: 285 (100), 241 (47), 151 (10)		
57	22.2	417	MS^2^ [417]: 399 (22), 357 (100), 327 (49)	Luteolin-*C*-pentoside	
			MS^3^ [417 → 357]: 339 (100), 311 (24), 297 (82), 285 (93)		
60	22.8	243	MS^2^ [243]: 225 (100), 201 (50), 199 (23), 157 (20)	Unknown	
75	32.1	485	MS^2^ [485]: 375 (100), 357 (13)	Unknown	
			MS^3^ [485 → 375]: 357 (100), 333 (22), 265 (39)		
79	36.0	285	MS^2^ [285]: 285 (100), 267 (5), 243 (2), 241 (3)	Luteolin	

Column "PVPP" indicate if the compound was also present in the corresponding *C. mariscus* treated PVPP sample.

**Table 7:** Characterization of the compounds present in the extract of *Limoniastrum monopetalum* aerial organs.

No.	Rt (min)	[M–H]^−^ *m/z*	m/z (% base peak)	Assigned identification	PVPP
1	1.8	377	MS^2^ [377]: 341 (100)	Disaccharide (HCl adduct)	+
			MS^3^ [377 → 341]: 179 (100), 161 (3), 143 (14), 119 (24), 113 (6)		
7	3.2	169	MS^2^ [169]: 125 (100)	Gallic acid	
9	4.6	305	MS^2^ [305]: 261 (21), 221 (53), 219 (57), 179 (100)	(Epi)gallocatechin	
12	7.2	305	MS^2^ [305]: 261 (17), 221 (32), 219 (49), 179 (100), 165 (25)	(Epi)gallocatechin	
16	8.6	303	MS^2^ [303]: 223 (100)	Sinapic acid sulfate	+
			MS^3^ [303 → 223]: 208 (100), 179 (37), 164 (35), 149 (5)		
24	10.2	273	MS^2^ [273]: 193 (100), 178 (17), 149 (38), 134 (7)	Ferulic acid sulfate	+
32	13.8	457	MS^2^ [457]: 329 (100), 169 (31)	Gallic acid derivative	+
			MS^3^ [457 → 169]: 125 (100)		
33	14.4	457	MS^2^ [457]: 329 (100), 245 (26), 203 (23), 165 (24)	Unknown	+
			MS^3^ [457 → 329]: 314 (100)		
46	19.1	252	MS^2^ [252]: 212 (100), 204 (4)	Unknown	
48	19.8	609	MS^2^ [609]: 301 (100)	Rutin	
			MS^3^ [609 → 301]: 179 (100), 151 (78)		
52	21.2	477	MS^2^ [477]: 301 (100)	Quercetin-*O*-glucuronide	
			MS^3^ [477 → 301]: 179 (90), 151 (100)		
55	21.7	567	MS^2^ [567]: 331 (100)	Unknown	
			MS^3^ [567 → 331]: 316 (100), 179 (67), 151 (33)		
64	24.1	437	MS^2^ [437]: 357 (100), 151 (52)	Pinoresinol	+
			MS^3^ [437 → 357]: 342(5), 311 (6), 151 (100), 136 (24)		
65	24.4	395	MS^2^ [395]: 315 (100)	Isorhamnetin sulfate	
			MS^3^ [395 → 315]: 300 (100), 271 (8), 255 (13)		
67	25.2	425	MS^2^ [425]: 345 (100)	Methylated flavonoid sulfate	
			MS^3^ [425 → 345]: 330 (100), 315 (34)		
			MS^4^ [425 → 345 → 330]: 315 (100), 285 (74)		
71	27.2	425	MS^2^ [425]: 345 (100), 330 (15)	Methylated flavonoid sulfate	
			MS^3^ [425 → 345]: 330 (100)		
			MS^4^ [425 → 345 → 330]: 315 (100), 271 (10)		
76	32.5	439	MS^2^ [439]: 359 (100)	Methylated flavonoid sulfate	+
			MS^3^ [439 → 359]: 344 (100)		
			MS^4^ [439 → 359 → 344]: 329 (100)		
80	36.9	439	MS^2^ [439]: 359 (100)	Methylated flavonoid sulfate	
			MS^3^ [439 → 359]: 344 (100), 329 (18)		
81	39.1	327	MS^2^ [327]: 291 (27), 229 (100), 211 (70), 209 (44), 171 (77)	Oxo-dihydroxy-octadecenoic acid	+
82	40.6	329	MS^2^ [329]: 311 (14), 229 (100), 211 (44), 171 (18)	Trihydroxy-octadecenoic acid	+

Column "PVPP" indicate if the compound was also present in the corresponding *L. monopetalum* treated PVPP sample.

The main constituents of *P. lentiscus* extract were flavonoid glycosides (mainly from myricetin and quercetin; approx. 53 mg g^−1^ DW), and galloylquinic acid and di-*O*-galloylquinic acid isomers (60 mg g^−1^ DW; Table 4; Suppl. files, Table I). In agreement to our findings, Romani et al. (2002) detected a high concentration of galloyl derivatives (5.3% DW), and a substantial amount of myricetin and quercetin glycosides (1.5% DW), extracted from a 70% ethanol solution of leaves^40^. Hydrolysable tannins are a group of gallic acid esters associated with polyols (e.g., glucose, glucitol, quinic acid), and the etherification or oxidation of the galloyl groups leads to the formation of complex structures (gallotannins and ellagitannins)^41^. Plant extracts containing hydrolysable tannins with gallic acid units were more effective as anthelmintics than those containing condensed tannins^42^. Nevertheless, the oligomerization and molecular weight of tannins may affect the anthelmintic activity, as is the case, for example, of elagitannins and condensed tannins^34,43^. Other metabolites present in lower concentrations in *P. lentiscus* extract with reported anthelmintic effects include flavan-3-ols and its galloyl derivatives, namely epigallocatechin (6.4 mg g^−1^ DW), gallocatechin gallate (6.8 mg g^−1^ DW) and catechin (5.0 mg g^−1^ DW). Molan et al. (2003) found that the presence of the galloyl group on flavan-3-ols was crucial for the activity on *T. colubrifomis* egg hatching (20% vs. 100% inhibition at 1 mg mL^−1^), and also more effective on immobilizing infective larvae (100% inhibition at 100–150 µg mL^−1^)^44^.

In *P. lentiscus* PVPP-treated samples, the concentration of flavonoid glycosides (0.17 mg g^−1^ DW) and galloylquinic acid (2.2 mg g^−1^ DW) drastically dropped (Suppl. files, Table I), which may justify the restoration of the egg hatching. On the other hand, the presence of these compounds in lower concentrations may explain the remaining activity on larvae. Nevertheless, compounds **2**, **58,** and **78** remained in this sample, and may also account for the activity.

Caffeoylquinic and dicaffeoylquinic acids were the most abundant compounds in *H. italicum picardi* extract (150 mg g^−1^ DW), followed by quercetin-*O*-glucosides (approx. 31 mg g^−1^ DW; Table 5; Suppl. files, Table II). These findings were expected, since previous works identified high contents of these metabolites in aerial organs of the same species^27,45^. Borges and colleagues (2019) found a significant correlation between the phenylpropanoid content (particularly chlorogenic acid, 1,3-dicaffeoylquinic, and 3,5-dicaffeoylquinic acids), and the ovicidal activity of 17 plant extracts from Pantanal wetlands against *Haemonchus placei*^46^*.* Additionally, chlorogenic acid exhibited an IC_50_ value of 92.4 μg mL^−1^ against L3 larvae exsheathment of *H. contortus*, and was also effective on preventing larvae hatching from eggs (IC_50_ = 520.8 μg mL^−1^)^47^. These results point out the potential of caffeoylquinic and dicaffeoylquinic acids to be the active metabolites of *H. italicum picardi* extracts. However, some *O*-glycosides are also present that may contribute to the detected activity. For example, Barrau and colleagues (2005) tested the activity of 3 flavonol glycosides (quercetin-3-*O*-rutinoside or rutin, kaempferol-3-rutinoside or nicotiflorin, and isorhamnetin-3-rutinoside or narcissin), and all reduced the migration of *H. contortus* L3 larvae in 25–35% when applied at 1200 µg mL^−1^^48^.

In *H. italicum picardi* PVPP-treated sample, although in lower concentrations, caffeoylquinic and dicaffeoylquinic acids remained in solution (8.3 mg g^−1^ DW), from which chlorogenic acid was the main compound (6.3 mg g^−1^ DW; Suppl. files, Table II). The high activity observed for the extract from *H. italicum picardi* treated with PVPP on larvae exsheathment is most likely due to the high content of chlorogenic acid remaining in the sample^47^. Still, other caffeoylquinic and dicaffeoylquinic acids are present (2 mg g^−1^ DW) that might also add to its effects. On the other hand, in EHIA the lower amount of these compounds in the PVPP-treated sample may have not be sufficient to inhibit egg hatching, since this process was completely restored. In fact, Borges and colleagues (2019) suggest that the concentration of monomeric and dimeric chlorogenic acid derivatives that enter in contact with eggs seems to be determinant for the activity, as observed for *Melanthera latifolia* ethanolic extract that had low concentrations of these compounds. and was considered inactive (up to 80% egg hatching at 50 mg mL^−1^)^46^.

*Cladium mariscus* acetone water extracts were previously reported as a rich source of polyphenols, particularly tannins by spectrophotometric methods, and chlorogenic, ferulic, and syringic acids were detected in higher amounts, through HPLC–DAD analysis^15,49^. In agreement, in this study, *C. mariscus* extract was mainly composed of flavan-3-ols (epigallocatechin, catechin), proanthocyanidins (5.1 mg g^−1^ DW), luteolin, C-glycosyl luteolin, a kaempferol glucoside, and an apigenin flavone (9.5 mg g^−1^ DW; Table 6; Suppl. files, Table III). Flavan-3-ols and proanthocyanidins have well recognized anthelmintic effects^44,50^, and therefore, they are most likely involved in the activity of *C. mariscus* extract. Also, the activity of the flavonoid luteolin on *H. contortus* larvae exsheathment has been previously established (IC_50_ = 17.1 and < 71.5 µM)^51^. Interestingly, Klongsiriwet and colleagues (2015) found that luteolin, even at low concentrations (30 μM), display synergistic effects with procyanidins, leading to a fivefold lower IC_50_ of the mixture in comparison to the procyanidin fraction alone (75.9 vs. 356 μg mL^−1^)^51^. Having this in mind, the combination of proanthocyanidins and luteolin in *C. mariscus* extract could act synergistically in the inhibition of the egg hatching. Nevertheless, the activity on LEIA was only partially restored after PVPP addition (approx. 20–40% larvae exsheathment), i.e., the remaining metabolites are still exhibiting anthelmintic properties. In PVPP-treated samples, mainly *C*-glycosyl flavones (1.07 mg g^−1^ DW) and, to a less extent chlorogenic acid, remained in solution while the catechin derivatives and luteolin were removed (Table 6; Suppl. files, Table III). As previously addressed, chlorogenic acid exhibits significant anthelmintic activity in vitro against *H. contortus* larvae exsheathment and egg hatching^47^. Despite the activity described for luteolin, the investigation of the anthelmintic properties of its glycosides is lacking. In general, *C*-glycosyl flavones exhibit antioxidant and anti-inflammatory properties^52^, and two flavone-*C*-glycosides namely isoschaftoside and schaftoside shown strong toxicity (LC_50_ = 114.66 μg mL^−1^ and 323.09 μg mL^−1^) against the plant-parasitic nematode *Meloidogyne incognita*^53^. Moreover, it is worth noticing that compounds **20** and **39** are still unidentified, although present in PVPP-treated samples.

Previous works identified several phenolic compounds in *L. monopetalum* extracts including gallic, vanillic, ferulic, syringic, p-hydroxybenzoic, protocatechuic, chlorogenic, and trans-cinnamic acids, and also quercetin, apigenin, amentoflavone, flavones, methyl gallate, and myricetin^54,55^. In the current work, the main metabolites identified in *L. monopetalum* extract were epigallocatechin, phenolic acids and derivatives, isorhamnetin sulfate, pinoresinol, methylated flavonoids sulfate and two oxylipins (Table 7). However, some of the major compounds, namely the methylated flavonoids sulfate **67**, **71**, **76**, and **80** were not identified, as well as the minor metabolites **33**, **46**, and **55**. The production of sulfated metabolites by plants is pointed out as an evolutionary trait to thrive in aquatic saline habitats, and part of the plant heavy metal detoxification mechanism^56,57^. Indeed, *L. monopetalum* is a halophytic and metal accumulator shrub that thrives in saltmarshes under harsh biotic and abiotic stresses (e.g., tidal fluctuations, salinity, heavy metal soils, sunlight exposure, UV radiation). Sulfated phenolics were previously identified in other halophyte species, such as *Limonium caspium* (Willd.) Gams^58^ and *Halimione portucaloides* (L.) Aellen^59^. The pharmacological interest in sulphated flavonoids increased in the last decades, mainly driven by its hydrophobic nature, and many reported biological activities, like anti-coagulant, anti-viral, antioxidant, anti-inflammatory, antimicrobial^60^.

Besides epigallocatechin (9.46 mg g^−1^ DW), the concentration of isorhamnetin sulfate (**65**) was high in *L. monopetalum* (6.4 mg g^−1^ DW) as well as phenolic acids and its derivatives (10.3 mg mL^−1^ DW; **7**, **16**, **24**, **32**). Delgado-Nuñez and colleagues (2020) attributed the main anthelmintic effects of *Prosopis laevigata* Willd. M. Johnston to isorhamnetin, which caused 100% of mortality on *H. contortus* eggs at the lowest concentration tested (700 µg mL^−1^), being also effective towards larvae (IC_50_ = 2.07 mg mL^−1^)^61^. The glycoside isorhamnetin-3-rutinoside decreased *H. contortus* L3 migration by 35% at 120 µg mL^−1^^48^. However, the activity of its sulfate structure is not reported. Among different classes of phenolic compounds, phenolic acids (i.e., caffeic acid, ferulic acid, and gallic acid) were the most potent anthelmintic metabolites against both *H. contortus* egg hatching (IC_50_ values = 0.56–4.93 µg mL^−1^) and larval development (IC_50_ = 22–33 µg mL^−1^)^62^. Nevertheless, one should keep in mind that structural modifications, such as glycosylation, methylation, and sulfation, may affect the bioactivity observed. For example, the substitution by a sugar unit in the quercetin structure showed a twofold increase in the larvicidal activity of rutin^62^. Still, studies concerning the anthelmintic effects of sulphated phenolics are missing. Since these metabolites are the main suspects as bioactive components of *L. monopetalum* extract, it would be interesting for further works to be conducted, not only confirming the anthelmintic effects of isolated compounds but also clarifying the role of sulfate in structure–activity relationship studies.

After PVPP treatment, the activity of *L. monopetalum* extract on larvae exsheathment was restored by approximately 60–70% to the control values. Although the remaining compounds may have contributed to the overall activity, the major anthelmintic effects were annulated. As some main metabolites of *L. monopetalum* (**67**, **71**, **76**, **80**) remain to be identified and quantified, further studies on this species are required to completely understand its bioactive compound (s) and related anthelmintic properties.

## Concluding remarks

Due to the constant diffusion of resistance to synthetic anthelmintics in worm populations, the search for plants with antiparasitic activities and their bioactive metabolites that can be used for integrated control approaches of GIN, has expanded over the last 20 years^63^. Extremophile plants, in particular salt-tolerant species, may represent an untapped reservoir of anthelmintic compounds for such purpose. To the best of our knowledge, this study explores for the first time the in vitro anthelmintic properties of eight salt-tolerant species, namely *H. italicum* subsp. *picardi*, *I. crithmoides*, *C. soldanela*, *C. mariscus*, *M. marina*, *P. coronopus*, *L. monopetalum*, and *C. maritima*, against two GIN species and life stages. *Pistacia lentiscus*, *L. monopetalum*, *C. mariscus*, and *H. italicum* subps. *picardi* were the most active against both parasite species and life stages (eggs and L3) targeted. The comparative HPLC-ESI-MS^n^ analysis coupled with the use of PVPP unraveled that different bioactive metabolites may be involved in the anthelmintic properties: flavonoid glycosides and galloylquinic acid isomers in *P. lentiscus*; caffeoylquinic and dicaffeoylquinic acids and quercetin glycosides in *H. italicum picardi*; proanthocyanins, phenolic acids, and luteolin in *C. mariscus*; and sulphated and/or methylated flavonoids in *L. monopetalum*. Further work should be pursued to complete the identification of the main metabolites of *L. monopetalum*, since this species exhibited the most promising results after *P. lentiscus*. As recently comprehensively reviewed by Spiegler et al.^65^ and Liu and colleagues^64^, polyphenols have been the most extensively studied compounds regarding their anthelmintic effects but the number of other individual phenolic compounds and their structural diversity investigated is still limited, particurlary towards these two GIN species. Therefore, future work should focus on fully elucidate the activity of the main potential bioactive metabolites identified in this work, either alone and/or in synergy, and provide information on structure–activity effects. Still, the results obtained in this study for *L. monopetalum*, *C. mariscus*, and *H. italicum* subsp. *picardi* warrant further investigations on the potential use of these species either as nutraceutical and/or phytotherapeutic options and/or as sources of anthelmintic compounds against GIN in ruminants.

## Supplementary Information

Supplementary Information.

## References

[1] Food and Agriculture Organization of the United Nations. *FAOSTAT Statistical Database*. Accessed http://www.fao.org/faostat/en/#data/QA on 13 April 2021.

[2] Hoste H, Sotiraki S, Landau SY, Jackson F, Beveridge I (2010). Goat–Nematode interactions: think differently. Trends Parasitol..

[3] Mavrot F, Hertzberg H, Torgerson P (2015). Effect of gastro-intestinal nematode infection on sheep performance: A systematic review and meta-analysis. Parasit. Vectors.

[4] Charlier J, Thamsborg SM, Bartley DJ, Skuce PJ, Kenyon F, Geurden T, Hoste H, Williams AR, Sotiraki S, Höglund J, Chartier C, Geldhof P, van Dijk J, Rinaldi L, Morgan ER, von Samson-Himmelstjerna G, Vercruysse J, Claerebout E (2018). Mind the gaps in research on the control of gastrointestinal nematodes of farmed ruminants and pigs. Transbound. Emerg. Dis..

[5] Besier RB, Kahnx LP, Sargison ND, Van Wykjj JA (2016). Diagnosis, treatment and management of *Haemonchus contortus* in small ruminants. Adv. Parasitol..

[6] Rose Vineer H, Morgan ER, Hertzberg H, Bartley DJ, Bosco A, Charlier J, Chartier C, Claerebout E, de Waal T, Hendrickx G, Hinney B, Hoglund J, Jezek J, Kasny M, Keane OM, Martínez-Valladares M, Munoz AM, Phythian CJ, Ploeger HW, Rataj AV, Skuce PJ, Simin S, Sotiraki S, Spinu M, Stuen S, Thamsborg SM, Vadlejch J, Varady M, von Samson-Himmelstjerna G, Rinaldi L (2020). Increasing importance of anthelmintic resistance in European livestock: Creation and meta-analysis of an open database. Parasite.

[7] Hoste H, Torres-Acosta JF, Sandoval-Castro CA, Mueller-Harvey I, Sotiraki S, Louvandini H, Thamsborg SM, Terrill TH (2015). Tannin containing legumes as a model for nutraceuticals against digestive parasites in livestock. Vet. Parasitol..

[8] Niezen JH, Robertson HA, Waghorn G, Charleston WA (1998). Production, faecal egg counts and worm burdens of ewe lambs which grazed six contrasting forages. Vet. Parasitol..

[9] Niezen JH, Waghorn GC, Charleston WAG (1998). Establishment and fecundity of *Ostertagia circumcincta* and *Trichostrongylus colubriformis* in lambs fed lotus (*Lotus pedunculatus*) or perennial ryegrass (*Lolium perenne*). Vet. Parasitol..

[10] Paolini V, Fouraste I, Hoste H (2004). In vitro effects of three woody plant and sainfoin on thrid-stage larvae and adult worms of three gastrointestinal nematodes. Parasitology.

[11] Zangueu CB, Olounlade AP, Ossokomack M, Djouatsa YNN, Alowanou GG, Azebaze AGB, Llorent-Martinez EJ, Córdova MLF, Dongmo AB, Hounzangbe-Adote MS (2018). In vitro effects of aqueous extract from *Maytenus senegalensis* (Lam.) Exell stem bark on egg hatching, larval migration and adult worms of *Haemonchus contortus*. BMC Vet. Res..

[12] Santos FO, Cerqueira APM, Branco A, Batatinha MJM, Botura MB (2019). Anthelmintic activity of plants against gastrointestinal nematodes of goats: A review. Parasitology.

[13] Le Houérou HN, Squires VR, Ayoub AT (1994). Forage halophytes and salt-tolerant fodder crops in the Mediterranean Basin. Halophytes as Resource for Livestock and for Rehabilitation of Degraded Lands. Tasks for Vegetation Science.

[14] Di Ferdinando M, Brunetti C, Agati G, Tattini M (2014). Multiple functions of polyphenols in plants inhabiting unfavorable Mediterranean areas. Environ. Exp. Bot..

[15] Lopes A, Rodrigues MJ, Pereira C, Oliveira M, Barreira L, Varela J, Trampetti F, Custódio L (2016). Natural products from extreme marine environments: Searching for potential industrial uses within extremophile plants. Ind. Crop. Prod..

[16] Oliveira M, Hoste H, Custódio L (2021). A systematic review on the ethnoveterinary uses of Mediterranean salt-tolerant plants: Exploring its potential use as fodder, nutraceuticals or phytotherapeutics in ruminant production. J. Ethnopharmacol..

[17] Piluzza G, Virdis S, Serralutzu F, Bullitta S (2015). Uses of plants, animal and mineral substances in Mediterranean ethno-veterinary practices for the care of small ruminants. J. Ethnopharmacol..

[18] Flowers, T., Santos, J., Jahns, M., Warburton, B., & Reed, P. *eHALOPH—Halophytes database*. Accessed https://www.sussex.ac.uk/affiliates/halophytes/ on 5 May 2021.

[19] Jallali I, Zaouali Y, Missaoui I, Smeoui A, Abdelly C, Ksouri R (2014). Variability of antioxidant and antibacterial effects of essential oils and acetonic extracts of two edible halophytes: *Crithmum maritimum* L. and *Inula crithmoiides* L. Food Chem..

[20] Singleton VL, Rossi JA (1965). Colorimetry of total phenolics with phosphomolybdic-phosphotungstic acid reagents. Am. J. Enol. Vitic..

[21] Quettier-Deleu C, Gressier B, Vasseur J, Dine T, Brunet C, Luyckx M, Cazin M, Cazin JC, Bailleul F, Trotin F (2000). Phenolic compounds and antioxidant activities of buckwheat (*Fagopyrum esculentum* Moench) hulls and flour. J. Ethnopharmacol..

[22] Li YG, Tanner G, Larkin P (1996). The DMACA–HC1 protocol and the threshold proanthocyanidin content for bloat safety in forage legumes. J. Sci. Food Agric..

[23] Rodrigues MJ, Soszynski A, Martins A, Rauter AP, Neng NR, Nogueira JMF, Varela J, Barreira L, Custódio L (2015). Unravelling the antioxidant potential and the phenolic composition of different anatomical organs of the marine halophyte *Limonium algarvense*. Ind. Crop. Prod..

[24] Jackson F, Hoste H, Vercoe PE, Makkar HPS, Schlink AC (2010). In vitro methods for the primary screening of plant products for direct activity against ruminant gastrointestinal nematodes. In vitro Screening of Plant Resources for Extra Nutritional Attributes in Ruminants: Nuclear and Related Methodologies.

[25] Bahuaud D, Martinez-Ortiz-de-Montellano C, Chauveau S, Prevot F, Torres-Acosta JFJ, Fouraste I, Hoste H (2006). Effects of four tanniferous plant extracts on the in vitro exsheathment of third-stage larvae of parasitic nematodes. Parasitology.

[26] Doner WL, Becard G, Irwin LP (1993). Binding of flavonoids by polyvinylpolypyrrolidone. J Agric Food Chem..

[27] Pereira CG, Barreira L, Bijttebier S, Pieters L, Neves V, Rodrigues MJ, Rivas R, Varela J, Custódio L (2017). Chemical profiling of infusions and decoctions of *Helichrysum italicum* subsp. *picardii* by UHPLC-PDA-MS and in vitro biological activities comparatively with green tea (*Camellia sinensis*) and rooibos tisane (*Aspalathus linearis*). J. Pharm. Biomed. Anal..

[28] Pereira CG, Custódio L, Rodrigues MJ, Neng NR, Nogueira JMF, Carlier J, Costa MC, Varela J, Barreira L (2017). Profiling of antioxidant potential and phytoconstituents of *Plantago coronopus*. Braz. J. Biol..

[29] Manolaraki F, Sotiraki S, Stefanakis A, Skampardonis V, Volanis M, Hoste H (2010). Anthelmintic activity of some Mediterranean browse plants against parasitic nematodes. Parasitology.

[30] Landau S, Azaizeh H, Muklada H, Glasser T, Ungar ED, Baram H, Abbas N, Markovics A (2010). Anthelmintic activity of *Pistacia lentiscus* foliage in two Middle Eastern breeds of goats differing in their propensity to consume tannin-rich browse. Vet. Parasitol..

[31] Azaizeh H, Halahleh F, Abbas N, Markovics A, Muklada H, Ungar ED, Landau SY (2013). Polyphenols from *Pistacia lentiscus* and *Phillyrea latifolia* impair the exsheathment of gastro-intestinal nematode larvae. Vet. Parasitol..

[32] Azaizeh H, Mrenya R, Markovics A, Muklada H, Glazer I, Landau SY (2015). Seasonal variation in the effects of Mediterranean plant extracts on the exsheathment kinetics of goat gastrointestinal nematode larvae. Small Rumin. Res..

[33] Brunet S, Hoste H (2006). Monomers of condensed tannins affect the larval exsheathment of parasitic nematodes of ruminants. J. Agric. Food Chem..

[34] Quijada J, Fryganas C, Ropiak HM, Ramsay A, Mueller-Harvey I, Hoste H (2015). Anthelmintic activities against *Haemonchus contortus* or *Trichostrongylus colubriformis* from small ruminants are influenced by structural features of condensed tannins. J. Agric. Food Chem..

[35] Mansfield LS, Gamble HR, Fetterer RH (1992). Characterization of the eggshell of *Haemonchus contortus*—I. Structural components. Comp. Biochem. Physiol. B Biochem. Mol. Biol..

[36] Oliveira AF, Costa Júnior LM, Lima AS, Silva CR, Ribeiro MNS, Mesquista JWC, Rocha CQ, Tangerina MMP, Vilegas W (2017). Anthelmintic activity of plant extracts from Brazilian savana. Vet. Parasitol..

[37] Zabré G, Kaboré A, Bayala B, Katiki LM, Costa-Júnior LM, Tamboura HH, Belem A, Abdalla AL, Niderkorn V, Hoste H, Louvandini H (2017). Comparison of the in vitro anthelmintic effects of *Acacia nilotica* and *Acacia raddiana*. Parasite.

[38] Vargas-Magaña JJ, Torres-Acosta JFJ, Aguilar-Caballero AJ, Sandoval-Castro CA, Hoste H, Chan-Pérez JI (2014). Anthelmintic activity of acetone–water extracts against *Haemonchus contortus* eggs: Interactions between tannins and other plant secondary compounds. Vet. Parasitol..

[39] Botura MB, dos Santos JDG, da Silva GD, de Lima HG, de Oliveira JVA, de Almeida MAO, Batatinha MJM, Branco A (2013). In vitro ovicidal and larvicidal activity of *Agave sisalana* Perr. (sisal) on gastrointestinal nematodes of goats. Vet. Parasitol..

[40] Romani A, Pinelli P, Galardi C, Mulinacci N, Tattini M (2002). Identification and quantification of galloyl derivatives, flavonoid glycosides and anthocyanins in leaves of *Pistacia lentiscus* L. Phytochem. Anal..

[41] Castañeda DM, Pombo LM, Urueña CP, Hernandez JF, Fiorentino S (2012). A gallotannin-rich fraction from *Caesalpinia spinosa* (Molina) Kuntze displays cytotoxic activity and raises sensitivity to doxorubicin in a leukemia cell line. BMC Complement. Altern. Med..

[42] Katiki LM, Ferreira JFS, Gonzalez JM, Zajac AM, Lindsay DS, Chagas ACS, Amarante AFT (2013). Anthelmintic effect of plant extracts containing condensed and hydrolyzable tannins on *Caenorhabditis elegans*, and their antioxidant capacity. Vet. Parasitol..

[43] Karonen M, Ahern JR, Legroux L, Suvanto J, Engström MT, Sinkkonen J, Salminen J-P, Hoste H (2020). Ellagitannins inhibit the exsheathment of *Haemonchus contortus* and *Trichostrongylus colubriformis* larvae: The efficiency increases together with the molecular size. J. Agric. Food Chem..

[44] Molan AL, Meagher LP, Spencer PA, Sivakumaran S (2003). Effect of flavan-3-ols on in vitro egg hatching, larval development and viability of infective larvae of *Trichostrongylus colubriformis*. Int. J. Parasitol..

[45] Gonçalves S, Moreira E, Grosso C, Andrade PB, Valentão P, Romano A (2017). Phenolic profile, antioxidant activity and enzyme inhibitory activities of extracts from aromatic plants used in Mediterranean diet. J. Food Sci. Technol..

[46] Borges DGL, Echeverria JT, Oliveira TL, Heckler RP, Freitas MG, Damasceno-Junior GA, Carollo CA, Borges FA (2019). Discovery of potential ovicidal natural products using metabolomics. PLoS ONE.

[47] Mancilla-Montelongo G, Castañeda-Ramírez GS, Torres-Acosta JFJ, Sandoval-Castro CA, Borges-Argáez R (2019). Evaluation of cinnamic acid and six analogues against eggs and larvae of *Haemonchus contortus*. Vet. Parasitol..

[48] Barrau E, Fabre N, Fouraste I, Hoste H (2005). Effect of bioactive compounds from Sainfoin (*Onobrychis viciifolia Scop*.) on the in vitro larval migration of *Haemonchus contortus*: Role of tannins and flavonol glycosides. Parasitology.

[49] Oliveira M, Rodrigues MJ, Neng NR, Nogueira JMF, Bessa RJB, Custódio L (2021). Seasonal variations of the nutritive value and phytotherapeutic potential of *Cladium mariscus* L. (Pohl.) targeting rumintant’s production. Plants.

[50] Molan AL, Sivakumaran S, Spencer PA, Meagher LP (2004). Green tea flavan-3-ols and oligomeric proanthocyanidins inhibit the motility of infective larvae of *Teladorsagia circumcincta* and *Trichostrongylus colubriformis* in vitro. Res. Vet. Sci..

[51] Klongsiriwet C, Quijada J, Williams AR, Mueller-Harvey I, Williamson E, Hoste H (2015). Synergistic inhibition of *Haemonchus contortus* exsheathment by flavonoid monomers and condensed tannins. Int. J. Parasitol. Drugs Drug Resist..

[52] Zeng P, Zhang Y, Pan C, Jia Q, Guo F, Li Y, Zhu W, Chen K (2013). Advances in studying of the pharmacological activities and structure–activity relationships of natural C-glycosylflavonoids. Acta Pharm. Sin. B.

[53] Du SS, Zhang HM, Bai CQ, Wang CF, Liu QZ, Liu ZL, Wang YY, Deng ZW (2011). Nematocidal flavone-C-glycosides against the root-knot nematode (*Meloidogyne incognita*) from *Arisaema erubescens* tubers. Molecules.

[54] Trabelsi N, Megdiche W, Ksouri R, Falleh H, Soumaya B, Hajlaoui H, Avdelly C (2010). Solvent effects on phenolic contents and biological activities of the halophyte *Limoniastrum monopetalum* leaves. LWT Food Sci. Technol..

[55] Bouzidi A, Baaka N, Salem N, Mhenni MF, Mighri Z (2016). *Limoniastrum monopetalum* stems as a new source of natural colorant for dyeing wool fabrics. Fibers Polym..

[56] Aquino RS, Grativol C, Mourão PAS (2011). Rising from the sea: Correlations between sulfated polysaccharides and salinity in plants. PLoS ONE.

[57] Cambrollé J, Mancilla-Leytón JM, Muñoz-Vallés S, Figueroa-Luque E, Luque T, Figueroa ME (2013). Evaluation of zinc tolerance and accumulation potential of the coastal shrub *Limoniastrum monopetalum* (L.) Boiss. Environ. Exp. Bot..

[58] Gadetskaya AV, Tarawneh AH, Zhusupova GE, Gemejiyeva NG, Cantrell CL, Cutler SJ, Ross SA (2015). Sulfated phenolic compounds from *Limonium caspium*: Isolation, structural elucidation, and biological evaluation. Fitoterapia.

[59] Vilela C, Santos SAO, Coelho D, Silva AMS, Freire CSR, Neto CP, Silvestre AJD (2014). Screening of lipophilic and phenolic extractives from different morphological parts of *Halimione portulacoides*. Ind. Crop. Prod..

[60] Correia-da-Silva M, Sousa E, Pinto MMM (2014). Emerging sulfated flavonoids and other polyphenols as drugs: Nature as an inspiration. Med. Res. Rev..

[61] Delgado-Núñez EJ, Zamilpa A, González-Cortazar M, Olmedo-Juárez A, Cardoso-Taketa A, Sánchez-Mendoza E, Tapia-Maruri D, Salinas-Sánchez DO, Mendoza-de Gives P (2020). Isorhamnetin: A nematocidal flavonoid from *Prosopis laevigata* leaves against *Haemonchus contortus* eggs and larvae. Biomolecules.

[62] Sprengel Lima C, Pereira MH, Gainza YA, Hoste H, Regasini LO, Chagas ACS (2021). Anthelmintic effect of *Pterogyne nitens* (Fabaceae) on eggs and larvae of *Haemonchus contortus*: Analyses of structure-activity relationships based on phenolic compounds. Ind. Crop. Prod..

[63] French KE (2018). Plant-based solutions to global livestock anthelmintic resistance. Ethnobiol. Lett..

[64] Liu M, Panda SK, Luyten W (2020). Plant-based natural products for the discovery and development of novel anthelmintics against nematodes. Biomolecules.

[65] Spiegler V, Liebau E, Hensel A (2017). Medicinal plant extracts and plant-derived polyphenols with anthelmintic activity against intestinal nematodes. Nat. Prod. Rep..

