# Supplementary material for: Disclosing the bioactive metabolites involved in the in vitro anthelmintic effects of salt-tolerant plants through a combined approach using PVPP and HPLC-ESI-MSn
Source: Sci Rep. 2021 Dec 21;11:24303. doi: 10.1038/s41598-021-03472-9 (PMC8692309; doi:10.1038/s41598-021-03472-9)
Supplement: Supplementary file 1 — Supplementary Information. [file 41598_2021_3472_MOESM1_ESM.docx]

**Supplementary Material**

# Disclosing the bioactive metabolites involved in the *in vitro* anthelmintic effects of salt-tolerant plants through a combined approach using PVPP and HPLC-ESI-MS^n^

# Marta Oliveira^1^, Caroline Sprengel Lima^2^, Setha Ketavong^3^, Eulogio J. Llorent-Martínez ^4^, Hervé Hoste ^3,5^ & Luísa Custódio ^1^*

Affiliations:

^1^ Centre of Marine Sciences, University of Algarve, Campus de Gambelas, 8005-139 Faro, Portugal

^2^ Laboratory of Antibiotics and Chemotherapeutics, São Paulo State University, IBILCE, S. José do Rio Preto, SP, Brazil.

^3^ INRA, UMR 1225 IHAP, 23 Chemin des Capelles, Toulouse F-31076, France

^4^ Department of Physical and Analytical Chemistry, Faculty of Experimental Sciences, University of Jaén, Campus Las Lagunillas, E-23071 Jaén, Spain

^5^ Université de Toulouse, ENVT, 23 Chemin des Capelles, Toulouse F-31076, France

*** Corresponding author:**

Luísa Custódio

Centre of Marine Sciences, Universidade do Algarve, *Campus* de Gambelas, 8005-139 Faro, Portugal

E-mail: [lcustodio@ualg.pt](mailto:lcustodio@ualg.pt)

**Abstract**

Strategies to reduce dependence on synthetic drugs for the treatment of gastrointestinal nematodes (GIN) infections in ruminants include the search for novel anthelmintic scaffolds on plants, yet salt-tolerant plants remain overlooked. This study aims to evaluate the *in vitro* anthelmintic properties of selected salt-tolerant plants against GIN, and identify the potential bioactive secondary metabolites involved. For that purpose, 80% acetone/water extracts were prepared from dried biomass of aerial organs of nine salt-tolerant plant species and tested against *Haemonchus contortus* and *Trichostrongylus colubriformis* by the Larval Exsheathment Inhibition Assay (LEIA) and Egg Hatching Inhibition Assay (EHIA). *Pistacia lentiscus, Limoniatrum monopetalum, Cladium mariscus* and *Helychrisum italicum picardi* were the most active in both GIN and life stages. To investigate the role of polyphenols in the anthelmintic activity, four selected extracts were treated with polyvinylpolypyrrolidone (PVPP), and non-treated and treated samples were further characterized by high-performance liquid chromatography with electrospray ionization mass spectrometric detection (HPLC-ESI-MS^n^). While polyphenols seem responsible for the EHIA properties, they are partially accountable to LEIA results. Several phenolics involved in the anthelmintic effects were identified and discussed. In sum, these species are rich sources of anthelmintic compounds and, therefore, are of major interest for nutraceutical and/or phytotherapeutic applications against GIN in ruminants.

**Keywords:** halophytes; *Haemonchus* sp.; *Trichostrongylus* sp.; nutraceuticals; phytotherapeutic products

**Characterization of compounds by HPLC-ESI-MS^n^**

The characterization of the phytochemicals was carried out by HPLC-ESI-MS^n^ using the negative ion mode, except for compound **58** (positive ion mode). Identification was performed using analytical standards – apigenin, caffeic acid, catechin, chlorogenic acid, ferulic acid, gallic acid, kaempferol, myricetin, neochlorogenic acid, procyanidin dimer B2, quercetin, rutin, and sinapic acid– as well as bibliographic information. Compounds were numbered by their retention time, keeping the same numbering in all samples. A brief explanation of the characterization follows.

*Flavonoids*

Most of the flavonoids found in the extracts were glycosylated flavonoids. They were characterized by the neutral losses of 308 Da (rutinoside), 176 Da (glucuronide), 162 Da (hexoside), 152 Da (gallate), 146 Da (deoxyhexoside) and 132 Da (pentoside). Aglycones were identified by comparison with analytical standards or bibliographic information.

Catechin (compound **17**) was identified by comparison with an analytical standard. Compound **28**, with the same fragmentation pattern, was thus identified as epicatechin. Several compounds were characterized as (epi)catechin derivatives. Compounds **9** and **12** were characterized as (epi)gallocatechin isomers, whereas compound **30** presented a fragmentation pattern consistent with (epi)gallocatechin gallate [1]. Compounds **11**, **19**, **21**, **22**, **23**, **25** and **27** were characterized as proanthocyanidin dimers and trimers [2,3]. Compounds **34**, **38**, **41**, **43**, **47** and **49** were myricetin glycosides. The aglycone myricetin was observed at *m/z* 317 (main fragment ions at *m/z* 271, 179 and 151). Compounds **45**, **48**, **50**, **51**, **52**, **61**, **66**, **70**, **72** and **77** were characterized as quercetin glycosides, depicting quercetin aglycone at *m/*z 301 (fragment ions at *m/z* 179 and 151). Compounds **29**, **53**, **62**, **73** and **74** were kaempferol glycosides. Kaempferol was observed in all cases at m/z 285 (comparison with an analytical standard). Compound **54**, with deprotonated molecular ion at *m/z* 493, suffered the neutral loss of 162 Da to yield mearnsetin at *m/z* 331 (main fragment ion at *m/z* 316) [4]. It was thus characterized as mearnsetin-*O*-hexoside. Compound **65** suffered the neutral loss of 80 Da (sulfate) to yield isorhamnetin at *m/z* 315 (main fragment at *m/z* 300). Compound **56** was identified as isorhamnetin-*O*-hexoside due to the loss of 162 Da to yield isorhamnetin at *m/z* 315. Compound **68** suffered the loss of 162 Da to yield apigenin at m/z 269 (comparison with an analytical standard), so it was characterized as apigenin-O-hexoside. Compounds **35** and **42**, with [M-H]^-^ at *m/z* 563, were characterized as apigenin-*C*-hexoside-*C*-pentoside isomers due to the fragment ions observed at [M-H-60]-, [M-H-90]-, [M-H-120]-, [M-H-180]-, and [M-H-210]-, characteristic of di-*C*-glycoside flavonoids [4]. Compound **79** was identified as luteolin by comparison with an analytical standard. Compounds **31**, **37**, **40** and **57** were luteolin-*C*-glycosides. Compound **37** was specifically identified as isoorientin due to the fragment ion at *m/z* 429 (absent in orientin) [5]. Compounds **67**, **71**, **76** and **80** exhibited similar fragmentation patterns: losses of 80 Da (sulfate) and 15 Da (methyl groups). They were tentatively characterized as methylated flavonoids, as they presented maximum UV wavelengths at approximately 350 nm, typical of flavonoids.

*Phenolic acids*

Compounds **3** and **8** displayed the neutral loss of 162 Da (hexoside) to yield the base peak at *m/z* 153, which was characterized as a dihydroxybenzoic acid (comparison with a protocatechuic acid analytical standard). Several caffeoylquinic acids (compounds **4**, **10** and **18**) and dicaffeoylquinic acids (compounds **44**, **59**, **63** and **69**) were characterized by using analytical standards and bibliographic information [6]. Compounds **16**, **24** and **26** were identified as hydroxycinnamic acids - sinapic acid, ferulic acid and caffeic acid, respectively – by comparison with analytical standards. For the characterization of compound **58**, we used the positive ion mode. The protonated molecular ion and fragmentation pattern were consistent with hydroferuloylglucose according to bibliographic data [7].

*Others*

Compound **1** suffered the neutral loss of 36 Da (HCl) to yield the base peak at *m/z* 341. Its fragmentation pattern was consistent with a disaccharide formed by two hexosides (probably glucose) [8]. Compound **2**, with deprotonated molecular ion at *m/z* 191, presented fragment ions at *m/z* 173 and 111. Although citric acid presents a similar fragmentation pattern, this compound was characterized as quinic acid after the analysis of a citric acid analytical standard (which showed a different retention time). Compound **7** was identified as gallic acid due to the [M-H]^-^ at *m/z* 169 and base peak at *m/z* 125. Several derivatives were also tentatively characterized. Compounds **6** presented fragment ions at *m/z* 191 (quinic acid) and 169/125 (gallic acid), so it was characterized as galloylquinic acid; with an additional 152 Da (gallic moiety), compounds **5**, **13** and **14** were characterized as di-*O*-galloylquinic acid isomers [10]. Compound **15** was characterized as methyl gallate [9] and **32** as a gallic acid derivative. Compound **64** was identified as pinoresinol by comparison of its experimental mass spectra with bibliographic data [11]. Compounds **81** and **82** were tentatively characterized as the lignans oxo-dihydroxy-octadecenoic and trihydroxy-octadecenoic acids [12].

**References**

1. Stewart, A.J., Mullen, W., Crozier, A. On-line high-performance liquid chromatography analysis of the antioxidant activity of phenolic compounds in green and black tea. *Mol. Nutr. Food Res.* **49**, 52-60. <https://doi.org/10.1002/mnfr.200400064> (2005).
2. Hamed, A.I., Al-Ayed, A.S., Moldoch, J., Piacente, S., Oleszek, W., Stochmal, A. Profiles analysis of proanthocyanidins in the argun nut (*Medemia argun* – an ancient Egyptian palm) by LC-ESI-MS/MS. *J. Mass Spectrom.* **49**, 306-315. <https://doi.org/10.1002/jms.3344> (2014).
3. Kajdžanoska, M., Gjamovski, V., Stefova, M. HPLC-DAD-ESI-MS^n^ identification of phenolic compounds in cultivated strawberries from Macedonia. *Maced. J. Chem. Chem. Eng.* **29**, 181-194. <https://doi.org/10.20450/mjcce.2010.165> (2010).
4. Han, J., Ye, M., Qiao, X., Xu, M., Wang, B.-R, Guo, D.-A. Characterization of phenolic compounds in the Chinese herbal drug *Artemisia annua* by liquid chromatography coupled to electrospray ionization mass spectrometry. *J. Pharm. Biomed. Anal.* **47**, 516–525. https://doi.org/10.1016/j.jpba.2008.02.013 (2008).
5. Algamdi, N., Mullen, W., Crozier, A. Tea prepared from *Anastatica hirerochuntica* seeds contains a diversity of antioxidant flavonoids, chlorogenic acids and phenolic compounds. *Phytochemistry*. **72**, 248–254. https://doi.org/10.1016/j.phytochem.2010.11.017. (2011).
6. Clifford, M.N., Knight, S., Kuhnert, N. 2005. Discriminating between the six isomers of dicaffeoylquinic acid by LC-MS^n^. *J. Agric. Food Chem.* **53**, 3821-3832. <https://doi.org/10.1021/jf050046h> (2005).
7. Ma, C., Xiao, S-y., Li, Z-g., Wang, W., Du, L-y. Characterization of active phenolic components in the ethanolic extract of *Ananas comosus* L. leaves using high-performance liquid chromatography with diode array detection and tandem mass spectrometry. *J. Chromatogr.* A **1165**, 39-44. https://doi.org/10.1016/j.chroma.2007.07.060 (2007).
8. Brudzynski, K., Miotto, D. Honey melanoidins: Analysis of the compositions of the high molecular weight melanoidins exhibiting radical-scavenging activity. *Food Chem.* **127**, 1023–1030. <https://doi.org/10.1016/j.foodchem.2011.01.075> (2011).
9. Li, J., Kuang, G., Chen, X., Zeng, R. 2016. Identification of chemical composition of leaves and flowers from *Paeonia rockii* by UHPLC-Q-Exactive Orbitrap HRMS. *Molecules*. **21**, 947. <https://doi.org/10.3390/molecules21070947> (2016).
10. Bastos, L.M., da Silva, F.M.A., de Souza, L.R.S., Sá, I.S.C., Mesquita, R.S., de Souza, A.D.L., Nunomura, R.C.S. Integrative approach based on simplex-centroid design, ESI-MS and chemometric analysis for comprehensive characterization of phenolic compounds from *Endopleura uchi* bark. *J. Braz. Chem. Soc.* **31**, 351-356 (2020).
11. Ye, M., Y. Yan, and D. Guo. Characterization of phenolic compounds in the Chinese herbal drug Tu-Si-Zi by liquid chromatography coupled to electrospray ionization mass spectrometry. *Rapid Commun. Mass Spectrom.* **19**, 1469–84. <https://doi.org/10.1002/rcm.1944> (2005).
12. Van Hoyweghen, L., De Bosscher, K., Haegeman, G., Deforce, D., Heyerick, A. *In vitr*o inhibition of the transcription factor NF-κB and cyclooxygenase by bamboo extracts. *Phytother. Res*. **28**, 224–230. <https://doi.org/10.1002/ptr.4978> (2014).

**Tables**

**Table I**. Quantification of the main compounds detected in *Pistacia lentiscus* extract, in non-treated (-) and treated-PVPP (+) samples.

| **Nº** | **Assigned identification** | **mg g^-1^ DW extract** | |
| --- | --- | --- | --- |
|  |  | **PVPP (-)** | **PVPP (+)** |
| *Flavonoids* |  |  |  |
| 9 | (Epi)gallocatechin | 6.4 ± 0.4 | ^--^ |
| 17 | Catechin | 5.0 ± 0.3 | -- |
| 30 | Gallocatechin gallate | 6.8 ± 0.4 | -- |
| 34 | Myricetin-Hex-gallate | 0.14 ± 0.01 | -- |
| 38+41+43 | Myricetin glycosides | 21 ± 1^a^ | 0.17 ± 0.01^b^ |
| 45 | Quercetin-Hex-gallate | 0.26 ± 0.02 | -- |
| 47+49 | Myricetin glycosides | 19 ± 1 | -- |
| 51 | Quercetin-*O*-Hex | 3.5 ± 0.2 | -- |
| 61+62 | Quercetin-Pen + Kaempferol-Hex | 2.2 ± 0.1 | -- |
| 66 | Quercetin-*O*-dHex | 6.0 ± 0.4 | -- |
| 70 | Quercetin-Pen-gallate | 0.76 ± 0.05 | -- |
| 73 | Kaempferol-*O*-dHex | 0.59 ± 0.04 | -- |
| 74 | Kaempferol-Pen-gallate | 0.24 ± 0.02 | -- |
| 79 | Luteolin | 1.13 ± 0.06 | -- |
| **Total** |  | **73 ± 2^a^** | **0.17 ± 0.01^b^** |
| *Others* |  |  |  |
| 6 | Galloylquinic acid | 15 ± 1^a^ | 2.2 ± 0.2^b^ |
| 13 | di-O-Galloylquinic acid | 21 ± 1 | -- |
| 14 | di-O-Galloylquinic acid | 8.3 ± 0.5 | -- |
| 15 | Methyl gallate | 16 ± 1 | -- |
| **Total** |  | **60 ± 2^a^** | **2.2 ± 0.2^b^** |
| **TIPC** |  | **133 ± 3^a^** | **2.4 ± 0.2^b^** |

Bold values represent the sum of each type of components. Different superscripts in the same line mean significant differences.

**Table II**. Quantification of the main compounds detected in *Helichrysum italicum picardi* extract, in non-treated (-) and treated-PVPP (+) samples.

| **Nº** | **Assigned identification** | **mg g^-1^ DW extract** | |
| --- | --- | --- | --- |
|  |  | **PVPP (-)** | **PVPP (+)** |
| *Phenolic acids* | |  |  |
| 8 | Dihydroxybenzoic acid-*O*- Hex | 0.27 ± 0.03^a^ | 0.21 ± 0.02^a^ |
| 10 | Neochlorogenic acid | 2.6 ± 0.2^a^ | 0.99 ± 0.07^b^ |
| 18 | Chlorogenic acid | 14.1 ± 0.7^a^ | 6.3 ± 0.4^b^ |
| 44 | Dicaffeoylquinic acid | 3.5 ± 0.2 | -- |
| 59 | Dicaffeoylquinic acid | 6.4 ± 0.3 | -- |
| 63 | Dicaffeoylquinic acid | 93 ± 4^a^ | 0.51 ± 0.03^b^ |
| 69 | Dicaffeoylquinic acid | 30 ± 2^a^ | 0.33 ± 0.02^b^ |
| **Total** |  | **150 ± 5^a^** | **8.3 ± 0.4^b^** |
| *Flavonoids* |  |  |  |
| 50 | Quercetin-*O*-Hex | 19 ± 1 | -- |
| 54 | Mearnsetin-*O*-Hex | 5.7 ± 0.4 | -- |
| 56 | Isorhamnetin-*O*-Hex | 1.7 ± 0.1 | -- |
| 61 | Quercetin-*O*-Pen | 8.1 ± 0.4 | -- |
| 72 | Quercetin-*O*-Hex | 1.6 ± 0.1 | -- |
| 77 | Quercetin-*O*-dHex-*O*-Hex | 1.9 ± 0.1 | -- |
| **Total** |  | **38 ± 1** | **--** |
| **TIPC** |  | **188 ± 5^a^** | **8.3 ± 0.4^b^** |

Bold values represent the sum of each type of components. Different superscripts in the same line mean significant differences.

**Table III**. Quantification of the main compounds detected in *Cladium mariscus* extract in non-treated (-) and treated-PVPP (+) samples.

| **Nº** | **Assigned identification** | **mg g^-1^ DW extract** | |
| --- | --- | --- | --- |
|  |  | **PVPP (-)** | **PVPP (+)** |
| *Catechin derivatives* | |  |  |
| 9 | (Epi)gallocatechin | 0.47 ± 0.03 | -- |
| 11+12 | Proanthocyanidin dimer+(epi)gallocatechin | 4.6 ± 0.3 | -- |
| **Total** |  | **5.1 ± 0.3** | **--** |
| *Flavonoids* |  |  | -- |
| 31 | Luteolin-*C*-Hex-*C*-Pen | 2.0 ± 0.1^a^ | 0.70 ± 0.05^b^ |
| 35 | Apigenin-*C*-Hex-*C*-Pen | 0.49 ± 0.03^a^ | 0.37 ± 0.03^b^ |
| 37 | Luteolin‐6‐*C*‐glucoside (isoorientin) | 2.0 ± 0.1 | -- |
| 53 | Kaempferol-*O*-Hex | 0.97 ± 0.06 | -- |
| 57 | Luteolin-*C*-Pen | 1.0 ± 0.07 | -- |
| 79 | Luteolin | 3.0 ± 0.2 | -- |
| **Total** |  | **9.5 ± 0.3^a^** | **1.07 ± 0.06^b^** |
| **TIPC** |  | **14.6 ± 0.4^a^** | **1.07 ± 0.06^b^** |

Bold values represent the sum of each type of components. Different superscripts in the same line mean significant differences.

**Table IV**. Quantification of the main compounds detected in *Limoniastrum monopetalum* extract, in non-treated (-) and treated-PVPP (+) samples.

| **Nº** | **Assigned identification** | **mg g^-1^ DW extract** | |
| --- | --- | --- | --- |
|  |  | **PVPP (-)** | **PVPP (+)** |
| *Phenolic acids* | |  |  |
| 7 | Gallic acid | 3.8 ± 0.2 | ^--^ |
| 16 | Sinapic acid sulfate | 0.37 ± 0.03^a^ | 0.33 ± 0.02^a^ |
| 24 | Ferulic acid sulfate | 0.83 ± 0.05^a^ | 0.46 ± 0.03^b^ |
| 32 | Gallic acid derivative | 5.3 ± 0.3^a^ | 0.65 ± 0.04^b^ |
| **Total** |  | **10.3 ± 0.4^a^** | **1.44 ± 0.05^b^** |
| *Flavonoids* |  |  |  |
| 9 | (Epi)gallocatechin | 8.6 ± 0.4 | -- |
| 12 | (Epi)gallocatechin | 0.86 ± 0.05 | -- |
| 65 | Isorhamnetin sulfate | 6.4 ± 0.3 | -- |
| **Total** |  | **15.9 ± 0.5** | **--** |
| **TIPC** |  | **26.2 ± 0.6^a^** | **1.44 ± 0.05^b^** |

Bold values represent the sum of each type of components. Different superscripts in the same line mean significant differences.
